# Initiating multi-strain probiotics is associated with clinically meaningful symptom relief, better quality of life, and fewer treatment escalations in IBS-D/IBS-M: a Chinese real-world propensity-matched cohort study

**DOI:** 10.3389/fmed.2026.1900112

**Published:** 2026-08-18

**Authors:** Zhengwo Jia, Xiaojiang Gu

**Affiliations:** Department of Gastroenterology, Tongxiang First People’s Hospital, Tongxiang, Zhejiang, China

**Keywords:** diarrhea-predominant irritable bowel syndrome, irritable bowel syndrome, probiotics, propensity score matching, quality of life, real-world evidence

## Abstract

**Inroduction:**

Evidence for probiotics in irritable bowel syndrome remains heterogeneous, although real-world use is common in Chinese gastroenterology practice. This study evaluated whether initiation of multi-strain probiotics, compared with contemporary standard care without probiotics, was associated with clinically meaningful symptom and quality-of-life improvement in adults with diarrhoea-predominant or mixed irritable bowel syndrome.

**Methods:**

This single-centre retrospective real-world cohort study identified adults with Rome IV IBS-D or IBS-M managed between January 2022 and December 2024 from electronic health records. A new-user design with a symmetrical 14-day landmark was used. After prespecified exclusions, 287 patients comprised the full analytic set, including 122 probiotic initiators and 165 non-users. One-to-one nearest-neighbour propensity score matching yielded 75 matched pairs. Outcomes were assessed at 4–6 and 8–12 weeks, with change in IBS Symptom Severity Score from baseline to 8–12 weeks as the primary endpoint.

**Results:**

In the matched cohort, probiotic initiation was associated with a greater reduction in IBS-SSS than standard care at 8–12 weeks, with mean changes of −95 versus −60 points and a mean difference of −35 points (95% CI: −50 to −20; p = 0.001). IBS-SSS response occurred more frequently with probiotics than with non-use (62.7% versus 44.0%), as did pain response (58.7% versus 40.0%) and adequate relief (54.7% versus 38.7%). IBS-QOL improved by 16.0 versus 8.0 points, and IBS-QOL minimal clinically important difference response was more frequent with probiotics (53.3% versus 34.7%). Adverse events were infrequent and similar between groups (16.0% versus 13.3%). Treatment escalation occurred less often with probiotics (24.0% versus 40.0%).

**Discussion:**

In routine Chinese hospital care, initiation of multi-strain probiotics was associated with earlier and clinically meaningful symptom improvement, better disease-specific quality of life, and fewer treatment escalations among adults with IBS-D or IBS-M, without an apparent excess of adverse events.

## 1 Introduction

Irritable bowel syndrome (IBS) is common, disabling, and costly for patients and health systems (1, 2). Recurrent abdominal pain accompanied by altered bowel habits impairs daily functioning and erodes health related quality of life (3), while frequent clinic visits, diagnostic testing, and unscheduled care contribute to substantial resource use (4). Among clinical subtypes, diarrhoea-predominant and mixed patterns account for a large share of encounters in gastroenterology practice and are characterised by loose or urgent stools, fluctuating symptom intensity, and a tendency towards treatment cycling when initial strategies provide only partial relief (5). In routine Chinese hospital care, these patients often re-attend within short intervals seeking symptom control that is durable and tolerable (2). A pragmatic, safe, and scalable option that can be layered onto existing regimens, therefore, has practical value even if the expected effect is moderate rather than transformative.

Current therapeutic options include antispasmodics to reduce cramping (6), loperamide to lessen stool frequency and urgency (7, 8), short courses of rifaximin for selected patients with diarrhoeal symptoms (9), bile acid sequestrants for suspected bile acid involvement (8), and low dose tricyclics or selective serotonin reuptake inhibitors to modulate pain processing and bowel function (10). Dietary strategies such as restriction of fermentable oligo-di-monosaccharides and polyols may help motivated patients who can access structured guidance (10). Despite this armamentarium, responses are heterogeneous and often incomplete, tolerability or access can limit use, and a meaningful proportion of patients progress to treatment escalation with multiple agents (7). For many clinicians and patients, the goal becomes not cure but steady, clinically perceptible improvement in pain, stool form, and daily functioning achieved with options that are safe, affordable, and acceptable over weeks to months.

There is biological plausibility that targeted manipulation of the gut microbiota could support these aims (11). Multi-strain probiotic combinations are hypothesised to act along complementary axes relevant to the pathophysiology of irritable bowel syndrome. These include modulation of microbial metabolites such as short chain fatty acids and lactic acid (12, 13). Reinforcement of epithelial barrier function, attenuation of low grade mucosal immune activation (14), and influence on sensory pathways implicated in visceral hypersensitivity and gut brain communication.

A further mechanism of interest in diarrhoeal phenotypes is bile salt hydrolase activity, which may deconjugate bile acids and alter the luminal bile acid pool that contributes to secretory drive and motility (15). Such pathways provide a coherent rationale for testing whether multi-strain products, used as adjuncts to standard care, can translate into patient-centred benefits that are perceptible within typical follow-up intervals.

The evidence base for probiotics in irritable bowel syndrome is mixed (16, 17). Trials differ by enrolled phenotype, strain composition, dose and duration, outcome selection, and adherence support, and many studies are small with short follow-up (16). Guideline statements generally acknowledge potential benefit for some patients but stop short of recommending routine use for all (17). In contrast, daily practice in Chinese hospitals frequently includes adjunctive multi-strain products for patients with troublesome diarrhoeal symptoms or fluctuating mixed patterns, often in combination with antispasmodics, loperamide, rifaximin, or bile acid sequestrants (18, 19). This divergence between cautious guidance and pragmatic adoption reflects a familiar gap in medicine (20). Randomised evidence sets a standard for internal validity under selected conditions, while clinicians must make decisions for broader populations with varied co-medication, imperfect adherence, and constraints that shape real-world care. Comparative real-world data that are methodologically careful and anchored to outcomes patients can feel are therefore needed to inform practice and to guide the design of definitive pragmatic trials.

Two further considerations inform the present study. First, patient-reported outcomes in functional gastrointestinal disorders should be interpreted in relation to minimal clinically important difference thresholds to ensure that statistical change corresponds to improvements patients can perceive (21, 22). The IBS Symptom Severity Score captures pain, distension, bowel dissatisfaction, and interference with life; a reduction of at least 50 points has been widely used as a threshold for clinically important improvement (22). Pain intensity reductions of at least 30% and the occurrence of adequate relief in at least half of study weeks have similar interpretability (22). Disease specific quality-of-life instruments complement symptom scores and reflect domains that matter to patients (21). Anchoring endpoints to these thresholds aligns analysis with decision-making at the bedside. Second, potential heterogeneity of treatment effect across recognisable clinical phenotypes is central for targeting. Diarrhoea-predominant patterns and higher baseline symptom burden may be more responsive to microbiome modulating strategies (23). Features suggestive of bile acid involvement, such as watery stools on waking or at night, postprandial urgency, a high proportion of Bristol types six or seven, or a history of cholecystectomy, may identify patients whose physiology is more amenable to interventions that influence bile acid handling (24, 25). Explicit assessment of these subgroups can move the field towards precision in use rather than indiscriminate adoption.

The methodological choices in this study were made to approximate practice while addressing key biases inherent to retrospective analyses. A new-user framework was adopted to align exposed and unexposed cohorts at a comparable initiation point, and a symmetrical 14-day landmark was applied to both groups so that early events did not distort comparisons.

Propensity score matching based on prespecified demographic, clinical, psychometric, treatment, utilisation, and calendar time variables was used to improve between group comparability and to target the average treatment effect on the treated, which is the population for whom clinicians consider probiotics in routine care. Assessment windows at 4 to 6 weeks and 8 to 12 weeks mirror typical follow-up rhythms in outpatient gastroenterology, permitting inferences that fit actual visit schedules. The primary endpoint was change in IBS Symptom Severity Score from baseline to the 8- to 12-week window, which is a continuous measure with established minimal clinically important difference (26, 27). Key secondary endpoints were selected for their clinical interpretability and included the symptom severity responder definition, a pain responder criterion, adequate relief in at least half of study weeks, and change in quality of life with a prespecified threshold for clinically important gain (28–30). Safety and a clinically relevant composite of treatment escalation encompassing add on pharmacotherapy, emergency department visits, and hospitalisation were also captured to reflect consequences that matter to patients and services.

Because clinicians ask which patients are most likely to benefit and when to judge continuation, the study prespecified stratified and nested comparisons that speak directly to these decisions. Analyses within diarrhoea-predominant and mixed subtypes and within categories of baseline severity were designed to describe variation in associations without invoking complex interaction models.

Two nested subsets were also examined. One identified patients with clinical features suggestive of bile acid involvement using information available in routine records (24, 31, 32). The other defined a high adherence subset using a medication possession ratio of at least 80% between initiation and the 8- to 12-week assessment. These analyses were specified to inform targeting and follow-up rather than to claim causal interactions, recognising that adherence is measured after treatment begins and therefore should be interpreted descriptively (33, 34).

Against this background, the present single-centre, propensity score-matched, real-world study sought to evaluate whether initiating a multi-strain probiotic, compared with contemporary standard care without probiotics, is associated with greater improvement in patient-centred outcomes over 8 to 12 weeks among adults with Rome IV irritable bowel syndrome of diarrhoea-predominant or mixed subtype in a Chinese hospital setting (19, 35). We further sought to determine whether the magnitude or consistency of associations differed by subtype and baseline severity and to explore whether recognisable clinical features suggestive of bile acid involvement or higher adherence were accompanied by higher observed responder proportions (36). By anchoring outcomes to minimal clinically important difference thresholds and by situating assessments within routine follow-up windows, the study aimed to generate evidence that is both interpretable for patients and actionable for clinicians (37). Finally, by capturing adverse events and a composite of treatment escalation, the study addressed not only symptom change but also the downstream consequences that influence quality of life and resource use (38).

The need for such evidence is considerable. Clinicians require signals that help them decide when to add a multi-strain probiotic to standard care, how long to continue before judging success or failure, and which patients to prioritise (16, 39). Patients benefit when expectations are grounded in realistic timelines and when measures of success are linked to thresholds they can feel (40). Health systems benefit when adjunctive options that are safe and inexpensive can modestly reduce the need for additional drugs and unscheduled care (19). At present, the literature provides an uneven map that mixes positive and neutral findings from diverse products under diverse conditions (16, 40). High-quality randomised trials will remain essential to define efficacy for specific formulations (39). In the meantime, methodologically careful real-world comparisons anchored to patient-centred outcomes can clarify whether the pragmatic use of multi-strain probiotics in Chinese hospital practice is associated with improvements that are meaningful to patients and relevant to service delivery and can identify subgroups in whom benefits appear more pronounced (41). The present study was designed to meet this need and to inform both everyday decisions and the planning of future enriched pragmatic trials.

## 2 Methods

### 2.1 Study design, setting, and data sources

#### 2.1.1 Study design and setting

We conducted a single-centre, retrospective, real-world cohort study at Tongxiang First People’s Hospital (Zhejiang, China), reported in accordance with STROBE. The study analysed de-identified routine-care data from the hospital-wide electronic medical record and hospital information system between 1 January 2022 and 31 December 2024. The protocol was approved by the Institutional Review Board of Tongxiang First People’s Hospital (approval number: 20241206); informed consent was waived given the retrospective design.

#### 2.1.2 Data sources, extraction, linkage, and quality control

Data were extracted from the hospital-wide electronic medical record and hospital information system, including gastroenterology outpatient records, emergency department encounters, diagnosis lists, electronic prescriptions, pharmacy dispensing records, laboratory/endoscopy reports, and follow-up notes. Scanned paper-based notes, when available, were used only to verify ambiguous information. Records were linked using the unique hospital patient identifier, visit identifier, prescription/dispensing dates, and index date. A standardised electronic case-report form was developed before extraction to capture demographics, IBS subtype, symptoms, bowel habit variables, comorbidities, concomitant medications, probiotic exposure, outcomes, adverse events, and treatment escalation. One investigator performed the initial extraction, and a second investigator verified eligibility, exposure classification, index date, and primary outcome data; discrepancies were adjudicated by a senior gastroenterologist. Before analysis, range checks, date-order checks, duplicate-record checks, and cross-checks between prescription and dispensing records were performed.

### 2.2 Participants, exposure, comparator, and index/landmark

Patients were identified through a two-step case-finding and diagnostic confirmation procedure. First, the hospital information system and electronic medical record were queried for adults with outpatient or emergency department diagnoses or diagnostic text terms consistent with irritable bowel syndrome during the study period, including ICD-10 K58.x codes and Chinese or English diagnostic terms corresponding to irritable bowel syndrome, diarrhoea-predominant IBS, mixed-type IBS, and IBS with diarrhoeal symptoms. Second, candidate records were manually reviewed to confirm Rome IV IBS, IBS-D or IBS-M subtype, symptom chronicity, baseline IBS-SSS availability, and the absence of documented organic gastrointestinal disease explaining diarrhoeal symptoms. The diagnosis used for analysis was therefore the confirmed Rome IV IBS-D or IBS-M diagnosis after chart review, rather than the initial HIS diagnosis alone. The inclusion criteria required age ≥18 years, confirmed Rome IV IBS-D or IBS-M, and availability of baseline and at least one follow-up assessment.

The exclusion criteria were prespecified: organic gastrointestinal disease, inflammatory bowel disease, or coeliac disease; acute infectious diarrhoea within 30 days; pregnancy or severe immunosuppression; concurrent faecal microbiota transplantation or other probiotic interventions; probiotic use within 90 days before index; or missing baseline or 8- to 12-week outcomes. The exposure was initiation of a routinely prescribed multi-strain probiotic preparation as part of usual care. New users had no probiotic prescription or dispensing record in the preceding 90 days and had documented probiotic use for at least 14 consecutive days after the index date. Product name, product type, formulation-level microbial composition, dosage strength, prescribed dose per administration, administration frequency, initial prescription duration, repeated dispensing, and total documented days supplied through the 8- to 12-week assessment were abstracted from electronic prescription and pharmacy dispensing records. In this hospital setting, routinely prescribed multi-strain probiotic preparations were approved oral formulary products containing combinations of *Bifidobacterium*, *Lactobacillus*, *Enterococcus*, *Bacillus*, and/or *Clostridium* species. Batch-level strain identifiers and batch-specific viable counts were not routinely captured in the electronic medical record; therefore, exposure analyses were conducted at the product/formulation and prescription-intensity level rather than as strain-specific efficacy analyses. Detailed exposure characteristics are reported in Supplementary Table S3. The comparator was contemporary standard care without probiotic prescription or dispensing during the index window. To mitigate immortal time bias, a new-user design with a 14-day landmark applied to both groups was used: The index date was probiotic initiation for exposed patients and a comparable visit date for non-users; both groups were required to remain at risk through day 14, and events before the landmark were not counted.

### 2.3 Outcomes and follow-up

Outcomes were abstracted at prespecified windows of 4–6 weeks and 8–12 weeks after index; when multiple assessments were available, the observation closest to the target week was selected. The primary outcome was change in IBS Symptom Severity Score from baseline to 8–12 weeks (ΔIBS-SSS; negative values indicate improvement; minimal clinically important difference [MCID] 50 points). Key secondary outcomes comprised IBS-SSS responder (≥50-point reduction), pain responder (≥30% reduction on the numeric rating scale), adequate relief (“yes” in ≥50% of study weeks), change in IBS-QOL, and IBS-QOL responder (≥14-point increase). Safety outcomes included bloating, worsening abdominal pain, discontinuation due to adverse events, and serious adverse events. Treatment escalation was defined as initiation of add-on pharmacotherapy (antispasmodics, loperamide, rifaximin, bile acid sequestrants, or low-dose tricyclics/SSRIs), emergency department visit, or hospitalisation for IBS-related complaints within 8–12 weeks.

### 2.4 Covariates and propensity score methods

**Figure 1:**
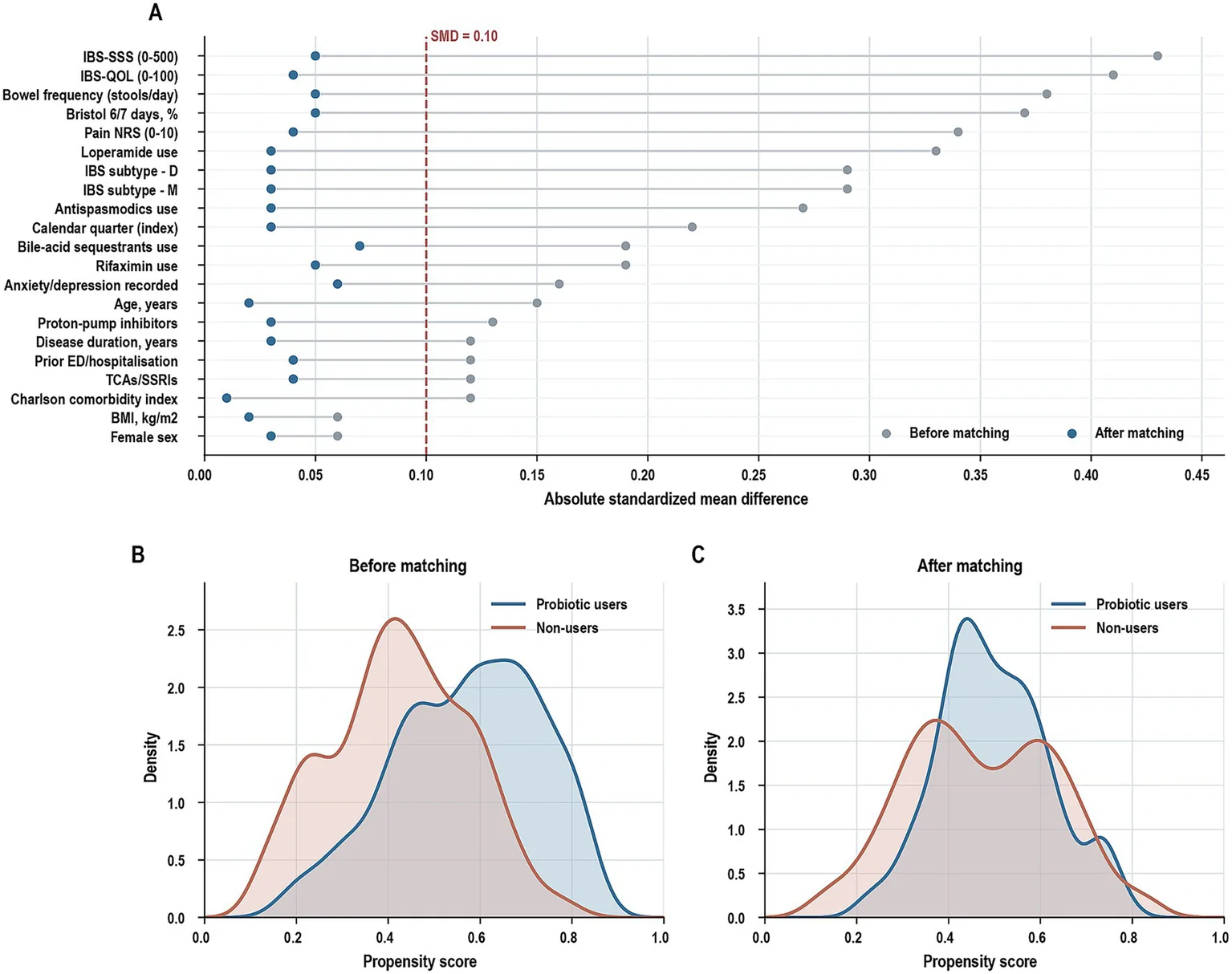
Propensity score matching diagnostics and covariate balance. Panel **(A)** shows absolute standardised mean differences for prespecified covariates before and after matching; The dashed line indicates the balance threshold of 0.10. Panel **(B)** presents the propensity score distributions of probiotic users and non-users before matching, and Panel **(C)** shows the corresponding distributions after matching. Matching substantially improved covariate balance and overlap between groups in the analytic framework of this study.

Prespecified covariates included age, sex, BMI, IBS subtype (D/M), disease duration, baseline IBS-SSS, pain NRS, IBS-QOL, Bristol stool types 6/7 proportion, bowel frequency, anxiety/depression measures, Charlson’s comorbidity index, prior emergency department visit or hospitalisation, concomitant medications around index (loperamide, antispasmodics, rifaximin, bile acid sequestrants, proton-pump inhibitors, and tricyclics/SSRIs), and calendar time (quarter). The Charlson comorbidity index was calculated from documented medical history and diagnosis codes recorded before or at the index visit using conventional Charlson’s weights. Age-adjusted Charlson’s scores were not used because age was entered separately as a prespecified covariate in the propensity score model. Component-level Charlson’s comorbidities are summarised in Supplementary Table S4. The suspected bile acid-related phenotype was defined as a clinical-criteria subset rather than a confirmed diagnosis of bile acid diarrhoea. This subset required at least two baseline features documented in routine records: watery stools on waking or at night, postprandial urgency, Bristol stool type 6 or 7 on at least 50% of abnormal bowel-movement days, prior cholecystectomy, or clinician-documented suspicion of bile acid-related diarrhoeal physiology. Patients with acute infection, inflammatory bowel disease, coeliac disease, colorectal malignancy, or other structural gastrointestinal disease had already been excluded before this phenotype was assigned. Serum 7α-hydroxy-4-cholesten-3-one, fibroblast growth factor 19, SeHCAT testing, and faecal bile acid quantification were not routinely available in this hospital setting; therefore, this phenotype was labelled as suspected rather than confirmed. A logistic regression estimated each patient’s propensity to initiate probiotics using all prespecified covariates. One-to-one nearest-neighbour matching without replacement was implemented with a 0.2 SD calliper on the logit of the propensity score under a common-support restriction. Balance was assessed using standardised mean differences (target |SMD| < 0.10) and variance ratios for continuous covariates; a Love plot summarised pre- and post-match covariate balance, and propensity score density plots were used to visualise overlap before and after matching (Figure 1). The matched estimand targets the average treatment effect on the treated (ATT).

### 2.5 Statistical analyses and robustness checks

All tests were two-sided with *α* = 0.05. Continuous outcomes were compared using Welch’s *t*-test and are presented as mean differences (MD) with 95% confidence intervals; Mann–Whitney tests and Hodges–Lehmann median differences were computed as sensitivity analyses. Binary outcomes were compared using χ^2^ or Fisher’s exact tests and are presented as proportions, absolute risk differences with Newcombe 95% confidence intervals, odds ratios with 95% confidence intervals, and numbers needed to treat (NNT = 1/absolute risk difference). Because matching induces within-pair dependence, inference was repeated with cluster-robust standard errors at the matched-pair level and within-pair permutation/bootstrap as robustness checks. Primary analyses followed an intention-to-treat principle within the matched set. In a per-protocol sensitivity analysis, patients who initiated probiotics in the control arm or discontinued in the exposed arm were censored at treatment change; the landmark was varied to 7 and 21 days with consistent direction of effects. For the primary matched analysis, patients were required to have baseline and 8- to 12-week IBS-SSS data; therefore, the primary endpoint was analysed in the complete-case matched cohort. Missing baseline or 8- to 12-week outcome data were part of the prespecified exclusion criteria. Missingness in baseline covariates used for propensity score estimation was low, with no single variable having more than 3% missingness; continuous covariates were handled by median imputation and categorical covariates by missing-category indicators before propensity score estimation. To assess whether missing follow-up outcomes could have influenced the findings, robustness checks were performed using inverse probability of censoring weighting and delta-adjusted pattern-mixture scenarios. These sensitivity analyses were interpreted as supportive rather than definitive because missingness reflected routine clinical documentation and follow-up practice. The missing-data pattern and robustness checks are summarised in Supplementary Table S5. Given the exploratory nature of secondary and stratified outcomes, no multiplicity adjustment was prespecified in the main text; Benjamini–Hochberg false discovery rate results are reported in Supplementary Table S6. To gauge susceptibility to unmeasured confounding, E-values were computed for the MCID responder odds ratio.

### 2.6 Sample size, ethics, and reproducibility

As a retrospective study, the sample size was determined by data availability. With 75 patients per group and an SD of approximately 40 points for ΔIBS-SSS, the study provides approximately 80% power to detect a 30- to 35-point between-group difference at *α* = 0.05, consistent with the established MCID. Analyses used R (version 4.3; MatchIt, cobalt, tableone, sandwich, ggplot2) and Python (version 3.10; matplotlib). Statistical code and analysis specifications are available from the corresponding author to support peer review. The ethics approval number was 20241206.

## 3 Results

### 3.1 Patient screening, exclusions, and construction of matched analytic cohorts

We screened 482 adults with Rome IV IBS between January 2022 and December 2024. Following predefined exclusions [organic gastrointestinal disease/IBD/coeliac disease, 32 (6.6%); acute infectious diarrhoea within 30 days, 21 (4.4%); pregnancy or severe immunosuppression, 11 (2.3%); concurrent faecal microbiota transplantation or other probiotic interventions, 9 (1.9%); missing baseline or 8- to 12-week outcomes, 84 (17.4%); prior probiotic use within 90 days, 38 (7.9%)], the final analytic cohort (FAS) comprised 287 patients (59.5% of those screened). Among the 84 patients excluded because of missing baseline or 8- to 12-week outcome data, missingness mainly reflected the absence of a documented IBS-SSS assessment within the prespecified 8- to 12-week follow-up window, rather than missing exposure or dispensing records. Within the FAS, 122 patients initiated multi-strain probiotics for ≥14 consecutive days as new users, and 165 received contemporary standard care without probiotics. Exposure details among probiotic initiators were traceable through electronic prescription and pharmacy dispensing records. The included probiotic preparations were routine hospital-formulary oral products prescribed according to labelled clinical dosing schedules, with formulation-level microbial composition, prescribed dose, administration frequency, initial prescription duration, repeated dispensing, total days supplied, and medication possession ratio summarised in Supplementary Table S3. Because formulation choice, dose, and duration were determined by routine clinical judgement rather than random assignment, analyses by treatment duration or adherence were interpreted as exploratory exposure-intensity analyses rather than causal dose–response analyses.

**Figure 2:**
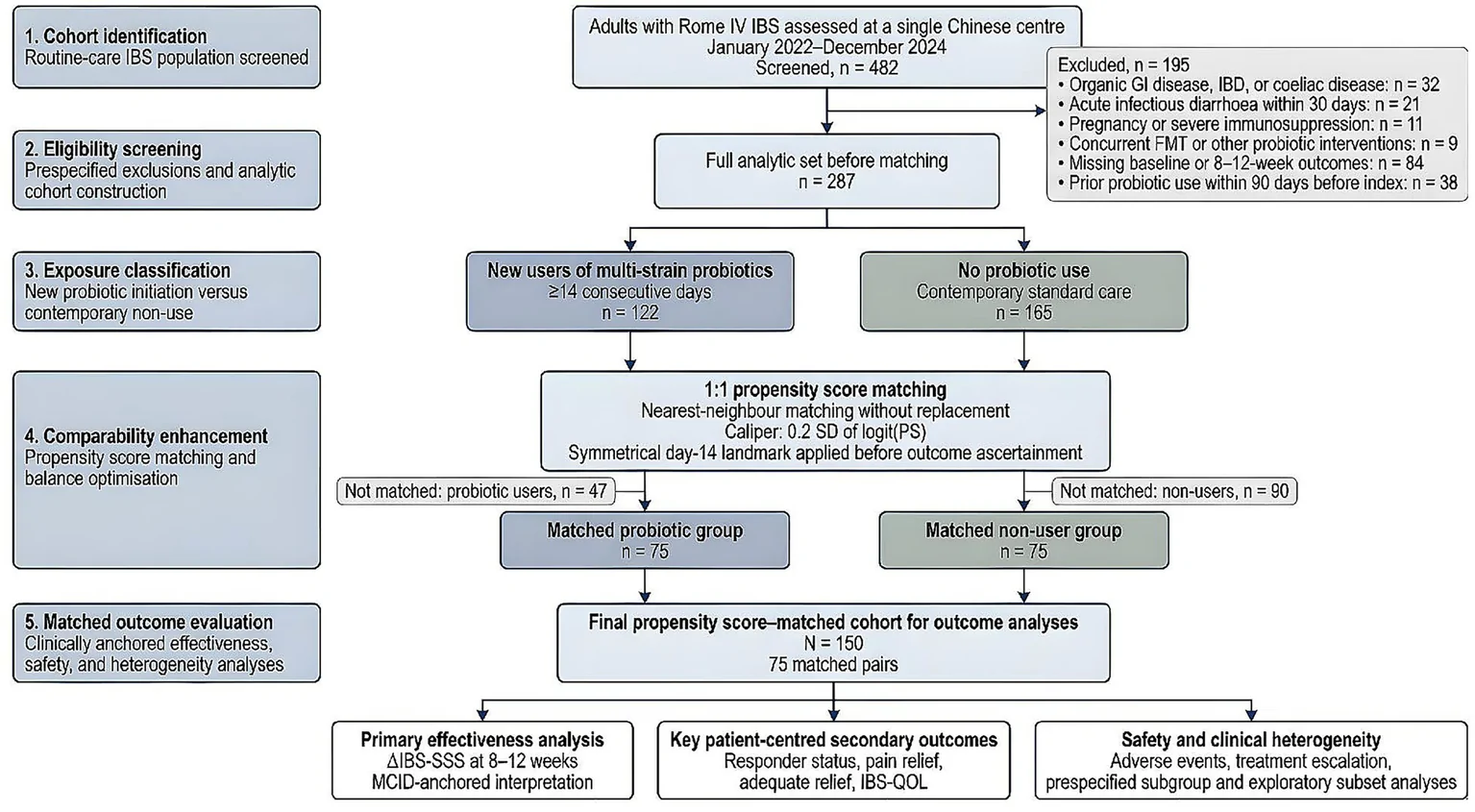
Study flow, propensity score matching, and analytic framework. Adults with Rome IV diarrhoea-predominant or mixed irritable bowel syndrome assessed between January 2022 and December 2024 were screened. After prespecified exclusions, 287 patients constituted the full analytic set before matching, including 122 new users of multi-strain probiotics and 165 non-users receiving contemporary standard care. One-to-one propensity score matching yielded 75 matched pairs for outcome analyses. The matched cohort was used to evaluate the primary effectiveness endpoint, key patient-centred secondary outcomes, safety, treatment escalation, and clinically relevant heterogeneity analyses.

Propensity score matching was conducted using one-to-one nearest-neighbour matching with a 0.2-SD calliper on the prespecified covariates (age, sex, IBS subtype [D/M], baseline IBS-SSS, pain NRS, IBS-QOL, BMI, anxiety/depression, Charlson’s index, prior ED/hospitalisation, concomitant medications, and calendar time) under a new-user design with a 14-day landmark. Lack of common support or missing covariates resulted in 47 probiotic users and 90 non-users remaining unmatched. The matched analysis set comprised 75 probiotic users and 75 non-users (*N* = 150) and was used for all primary outcome analyses; the FAS was retained for descriptive and sensitivity analyses (Figure 2).

### 3.2 Baseline imbalances in symptom burden resolved through propensity score matching

Before matching, 287 adults constituted the full analytic set, including 122 new users of multi-strain probiotics and 165 non-users. Several prespecified covariates differed meaningfully between groups. Probiotic users had greater baseline symptom burden and more diarrhoea-predominant phenotype: mean IBS-SSS 334 versus 306 (|SMD| 0.43), pain NRS 6.3 versus 5.8 (0.34), IBS-QOL 52.1 versus 57.2 (0.41), Bristol 6/7 days 52.4% versus 46.9% (0.37), bowel frequency 2.8 versus 2.4 stools/day (0.38), and IBS-D 68.0% versus 53.9% (0.29). The use of anti-diarrhoeals and antispasmodics around baseline was also more frequent among probiotic users, 32.0% versus 18.2% (0.33) and 41.0% versus 27.9% (0.27), respectively. Smaller imbalances were observed for age (41.8 versus 43.7 years; 0.15), disease duration (0.12), rifaximin (0.19), bile acid sequestrants (0.19), and calendar quarter at index (0.22). Other demographics, comorbidity burden, and psychometric markers showed modest or negligible differences (Supplementary Table S1).

Propensity score matching produced a cohort of 150 patients, such as 75 probiotic users and 75 non-users, in whom all prespecified covariates satisfied the balance target of |SMD| < 0.10. Post-match central tendencies were closely aligned: age: 42.1 versus 42.4 years (|SMD| 0.02), female sex: 58.7% versus 57.3% (0.03), BMI: 22.9 versus 22.8 kg/m^2^ (0.02), IBS-SSS: 318 versus 315 (0.05), pain NRS: 6.1 versus 6.0 (0.04), IBS-QOL: 54.6 versus 55.1 (0.04), Bristol 6/7 days: 49.0% versus 48.3% (0.05), and bowel frequency: 2.6 versus 2.5 stools/day (0.05). Comorbidity and prior utilisation were comparable, including Charlson’s index 0.7 versus 0.7 (0.01) and prior emergency department visit or hospitalisation 13.3% versus 14.7% (0.04). Component-level Charlson’s comorbidities were infrequent and balanced between groups; their distribution is shown in Supplementary Table S4. Concomitant medications around baseline were well balanced, for example, loperamide: 24.0% versus 22.7% (0.03), antispasmodics: 34.7% versus 33.3% (0.03), rifaximin: 8.0% versus 6.7% (0.05), bile acid sequestrants: 4.0% versus 2.7% (0.07), PPIs: 26.7% versus 28.0% (0.03), and TCAs/SSRIs: 13.3% versus 12.0% (0.04). Calendar time at index was evenly distributed across quarters. The Love plot visually confirmed covariate balance, with median post-match |SMD| 0.03 and maximum 0.07 (Figure 1). Detailed matched values are shown in the baseline table (Table 1).

**Table 1:** Baseline characteristics after propensity score matching (*N* = 150).

Variable	Probiotic users (*n* = 75)	Non-users (*n* = 75)	SMD
Demographics			
Age, years	42.1 (12.0)	42.4 (12.3)	0.02
Female, *n* (%)	44 (58.7)	43 (57.3)	0.03
BMI, kg/m^2^	22.9 (3.1)	22.8 (3.0)	0.02
Disease profile			
IBS subtype—D, *n* (%)	47 (62.7)	46 (61.3)	0.03
IBS subtype—M, *n* (%)	28 (37.3)	29 (38.7)	0.03
Disease duration, years, median (IQR)	3.0 (1.2–6.0)	3.0 (1.1–5.8)	0.03
IBS-SSS (0–500)	318 (64)	315 (65)	0.05
Pain NRS (0–10)	6.1 (1.3)	6.0 (1.4)	0.04
IBS-QOL (0–100)	54.6 (12.5)	55.1 (12.3)	0.04
Bowel habit			
Bristol 6/7 days, %	49.0 (14.8)	48.3 (14.6)	0.05
Bowel frequency, stools/day	2.6 (1.0)	2.5 (1.0)	0.05
Comorbidity & prior use			
Charlson’s comorbidity index	0.7 (0.8)	0.7 (0.8)	0.01
Anxiety/depression recorded, *n* (%)	20 (26.7)	18 (24.0)	0.06
Prior ED visit or hospitalisation, *n* (%)	10 (13.3)	11 (14.7)	0.04
Concomitant medications (baseline ±14 days)			
Loperamide (anti-diarrhoeal), *n* (%)	18 (24.0)	17 (22.7)	0.03
Antispasmodics, *n* (%)	26 (34.7)	25 (33.3)	0.03
Bile acid sequestrants, *n* (%)	3 (4.0)	2 (2.7)	0.07
Rifaximin, *n* (%)	6 (8.0)	5 (6.7)	0.05
Proton-pump inhibitors, *n* (%)	20 (26.7)	21 (28.0)	0.03
TCAs/SSRIs, *n* (%)	10 (13.3)	9 (12.0)	0.04
Calendar time at index			
Quarter 1, *n* (%)	19 (25.3)	18 (24.0)	0.03
Quarter 2, *n* (%)	19 (25.3)	19 (25.3)	0.00
Quarter 3, *n* (%)	19 (25.3)	19 (25.3)	0.00
Quarter 4, *n* (%)	18 (24.0)	19 (25.3)	0.03

Values are mean (SD) or *n* (%) unless stated. SMD, standardised mean difference; IBS-SSS, Irritable Bowel Syndrome–Symptom Severity Score (0–500); IBS-QOL, IBS–quality of life (0–100); NRS, numeric rating scale; BMI, body mass index. Prespecified balance target: |SMD| < 0.10. Baseline is defined as the index date (first probiotic prescription for users; matched index date for non-users). Concomitant medications were captured within ±14 days of baseline. Bristol 6/7 days (%) was calculated among abnormal bowel movement days per Rome IV guidance. Disease duration is presented as median (IQR); SMD for skewed variables was approximated using pooled-variance or rank-based standardisation. All prespecified covariates achieved the balance threshold of |SMD| < 0.10 after 1:1 nearest-neighbour matching with a 0.2-SD calliper. This matched cohort (*n* = 150) was used for primary efficacy analyses; the full analytic set and pre-match characteristics are provided in Supplementary Table S1. The Charlson comorbidity index was calculated from documented medical history and diagnosis codes recorded before or at the index visit using conventional Charlson’s weights; component-level comorbidities are provided in Supplementary Table S4.

### 3.3 Greater reduction in IBS symptom severity score with probiotics versus standard care

**Figure 3:**
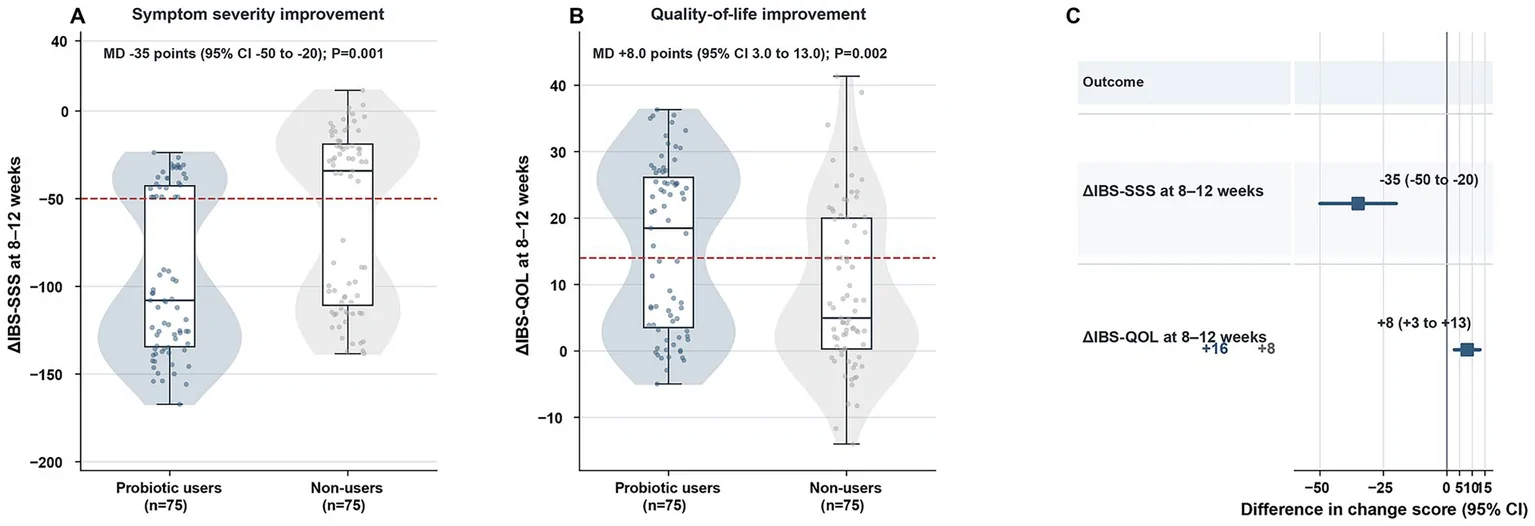
Primary symptom and quality-of-life improvement in the matched cohort. Panel **(A)** shows the distribution of change in IBS Symptom Severity Score (ΔIBS-SSS) from baseline to 8–12 weeks among propensity score–matched probiotic users and non-users. Negative values indicate symptom improvement, and the dashed line denotes the minimal clinically important difference (MCID, −50 points). Panel **(B)** shows the corresponding distribution of change in IBS–Quality of Life (ΔIBS-QOL) score; positive values indicate improvement, and the dashed line denotes the MCID threshold of +14 points. Violin plots display the overall distribution, boxplots indicate the median and interquartile range, and individual points represent patient-level values. Panel **(C)** summarises the between-group mean differences and 95% confidence intervals for both continuous outcomes. Probiotic users showed greater improvement in symptom severity and disease-specific quality of life than non-users.

**Figure 4:**
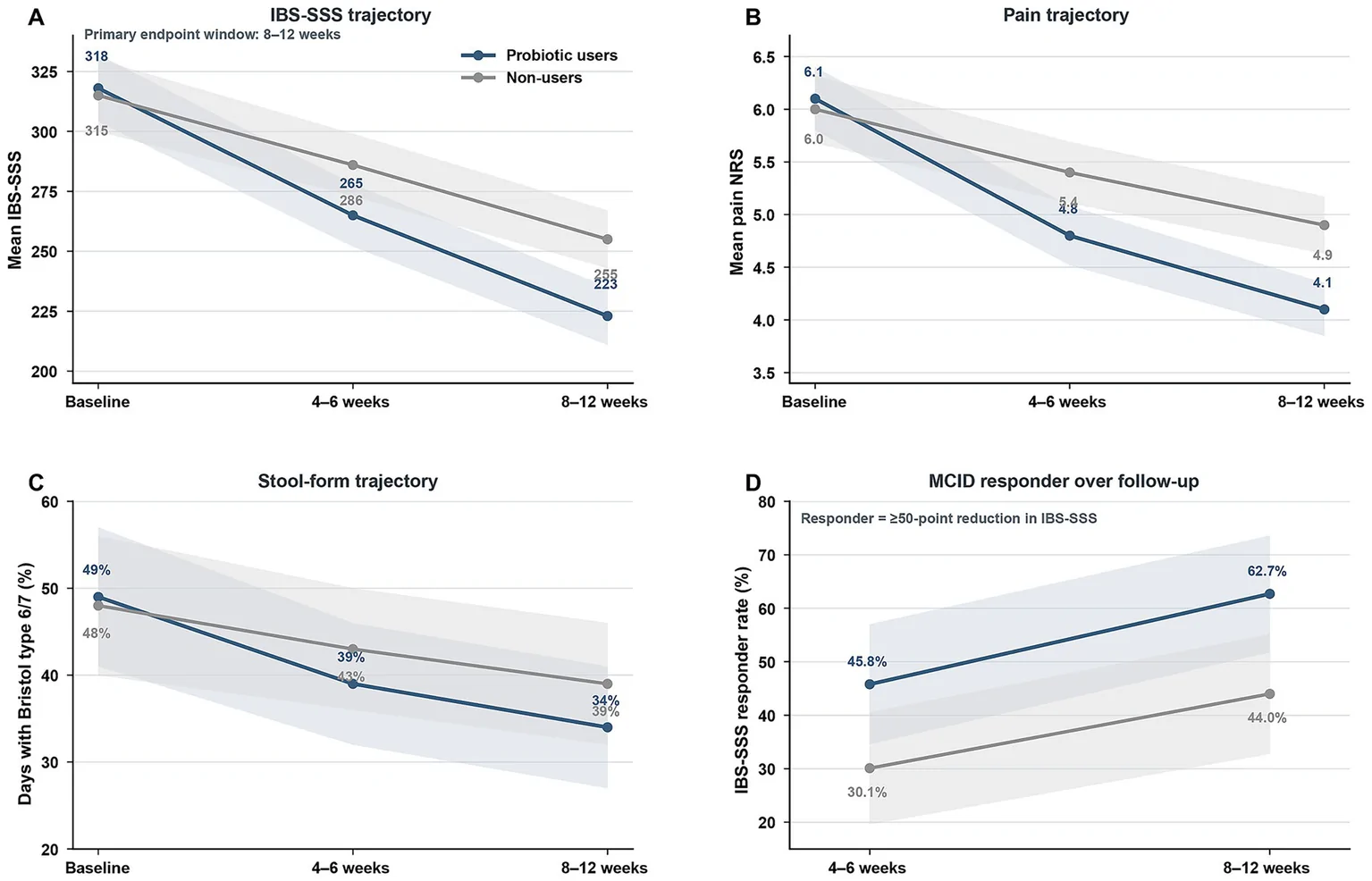
Time-resolved symptom and bowel habit trajectories in the matched cohort. Panel **(A)** shows the trajectory of mean IBS Symptom Severity Score (IBS-SSS) from baseline through 4–6 weeks and 8–12 weeks in propensity score–matched probiotic users and non-users. Panel **(B)** shows the corresponding trajectory for pain numeric rating scale (NRS), and panel **(C)** shows the proportion of days with Bristol stool types 6/7. Panel **(D)** shows the proportion of patients achieving an IBS-SSS responder status, defined as a reduction of at least 50 points from baseline, at 4–6 weeks and 8–12 weeks. Across these measures, probiotic users showed an earlier and more sustained pattern of improvement than non-users, with clearer separation by 8–12 weeks. Shaded bands indicate approximate 95% confidence intervals.

In the matched cohort of 150 patients, the primary endpoint of change in IBS Symptom Severity Score at 8–12 weeks showed greater improvement in probiotic users compared with non-users. The mean reduction in IBS-SSS was −95 (SD 40) in probiotic users versus −60 (SD 40) in non-users, corresponding to a mean difference of −35 (95% CI: −50 to −20, *p* = 0.001) (Table 2). Violin–box plots showed a broader distribution of symptom improvement in probiotic users, with more patient-level values extending beyond the prespecified minimal clinically important difference threshold of −50 points; the corresponding effect summary confirmed a between-group mean difference of −35 points (95% CI: −50 to −20) (Figures 3A,C). Line plots of IBS-SSS means with 95% confidence intervals further illustrated separation between groups by 4–6 weeks, with maximal divergence at 8–12 weeks (Figure 4A). Sensitivity analyses using non-parametric tests yielded similar estimates, supporting the robustness of the primary symptom-severity finding in the matched cohort (Table 2). Additional missing-data robustness checks were directionally consistent with the primary analysis, although estimates were attenuated under conservative missing-data assumptions (Supplementary Table S5).

**Table 2:** Primary outcome: change in IBS-Symptom Severity Score (ΔIBS-SSS) at 8–12 weeks after matching.

Subgroup	Probiotic users (n)	ΔIBS-SSS, mean (SD)	Non-users (n)	ΔIBS-SSS, mean (SD)	MD (95% CI)	*p*-value
Overall (matched set)	75	−95 (40)	75	−60 (40)	−35 (−50 to −20)	0.001
IBS-D subtype	47	−100 (42)	46	−65 (39)	−35 (−53 to −17)	0.002
IBS-M subtype	28	−88 (38)	29	−55 (37)	−33 (−54 to −12)	0.004
Per-protocol (MPR ≥ 80%)	62	−98 (39)	63	−61 (39)	−37 (−52 to −22)	<0.001

Values are mean (SD) unless otherwise stated. MD, mean difference (probiotic users minus non-users); CI, confidence interval; PP, per-protocol defined as medication possession ratio ≥80%. ΔIBS-SSS was defined as IBS-SSS at 8–12 weeks minus baseline; negative values indicate improvement. The primary endpoint was prespecified as group mean difference in ΔIBS-SSS at 8–12 weeks in the matched cohort. Estimates were obtained from independent-sample t-tests; the results were consistent with non-parametric tests (not shown). Subgroup analyses by IBS subtype (D, M) and per-protocol set are descriptive; no statistical interaction testing was performed. Clinically meaningful improvement was defined as a reduction ≥50 points, assessed in subsequent responder analyses.

### 3.4 Higher responder rates and quality-of-life gains in probiotic users

**Figure 5:**
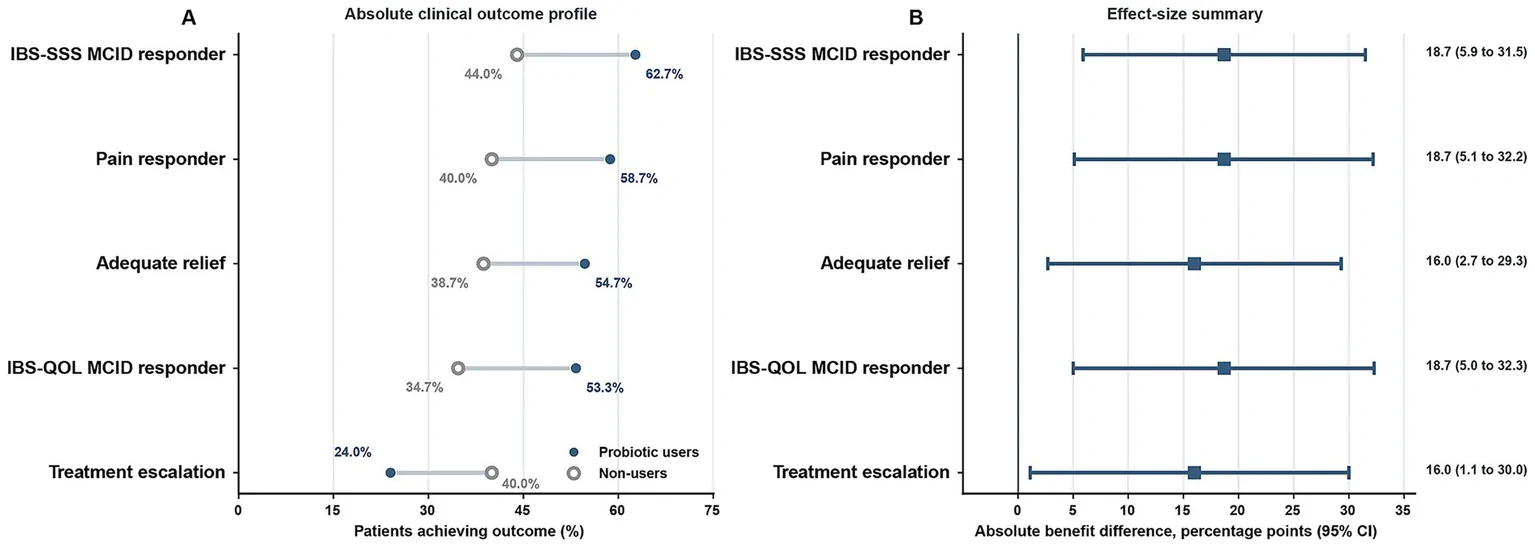
Patient-centred response profile and treatment-management benefit in the matched cohort. Panel **(A)** shows the absolute proportions of matched probiotic users and non-users achieving key clinical outcomes at 8–12 weeks, including IBS-SSS MCID response, pain response, adequate relief, IBS-QOL MCID response, and treatment escalation. Panel **(B)** summarises the corresponding absolute benefit differences and 95% confidence intervals. Positive values consistently favour probiotics; for treatment escalation, the effect is displayed as the absolute reduction versus non-users. Overall, probiotic users demonstrated higher rates of clinically meaningful response across multiple patient-centred outcomes and a lower likelihood of treatment escalation.

**Figure 6:**
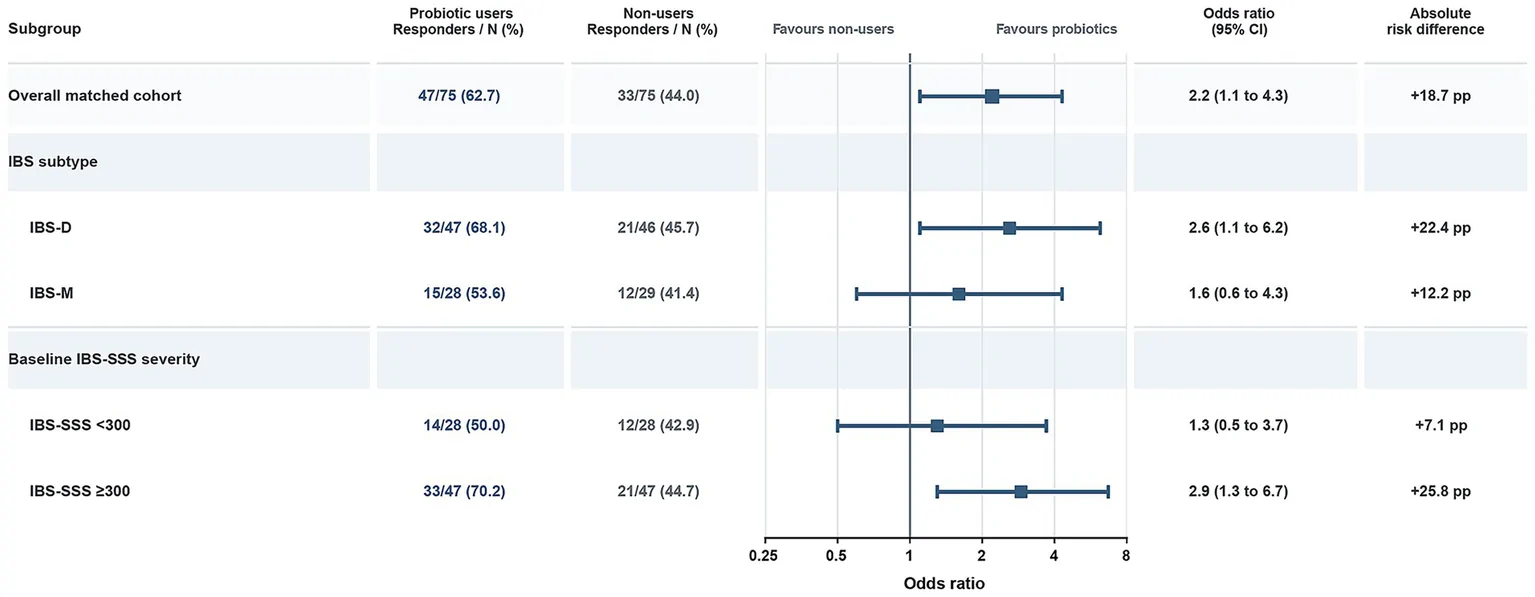
Prespecified clinical heterogeneity of IBS-SSS responder benefit in the matched cohort. Forest plot showing odds ratios and 95% confidence intervals for achieving an IBS-SSS MCID response, defined as a reduction of at least 50 points at 8–12 weeks, in the overall matched cohort and prespecified clinical strata. Subgroups included IBS subtype and baseline IBS-SSS severity. Responder counts and proportions for probiotic users and non-users are shown alongside the forest plot, together with absolute risk differences. Estimates favoured probiotics overall, with numerically larger associations in patients with IBS-D and in those with baseline IBS-SSS ≥ 300. Subgroup analyses were descriptive and not intended to establish formal interaction effects.

In the matched cohort, key patient-centred secondary outcomes were concordant with the primary endpoint. The proportion achieving the prespecified IBS-SSS responder definition of a reduction of at least 50 points was higher with probiotics than with standard care, 62.7% versus 44.0% (absolute risk reduction: 18.7 percentage points, 95% CI: 5.9 to 31.5; number needed to treat: 5.3, 95% CI: 3.2 to 16.9; odds ratio: 2.2, 95% CI: 1.1 to 4.3) (Table 3). Absolute response proportions and the corresponding absolute benefit differences across key patient-centred outcomes are summarised in Figures 5A,B. The prespecified subgroup forest plot showed a directionally consistent responder advantage for probiotic users across the overall matched cohort, IBS subtype strata, and baseline symptom-severity strata (Figure 6). Detailed stratum-specific estimates are presented in Section 3.6.

**Table 3:** Responder rates and effect estimates in the matched cohort.

Subgroup	Probiotic users	Non-users	ARR, percentage points	95% CI for ARR	NNT (95% CI)	OR (95% CI)
Overall	47/75 (62.7)	33/75 (44.0)	18.7	5.9 to 31.5	5.3 (3.2–16.9)	2.2 (1.1–4.3)
IBS-D subtype	32/47 (68.1)	21/46 (45.7)	22.4	6.1 to 38.7	4.5 (2.6–16.4)	2.6 (1.1–6.2)
IBS-M subtype	15/28 (53.6)	12/29 (41.4)	12.2	−10.7 to 35.1	NE	1.6 (0.6–4.3)
Per-protocol (MPR ≥ 80%)	42/62 (67.7)	28/63 (44.4)	23.3	8.1 to 38.5	4.3 (2.6–12.3)	2.7 (1.2–6.1)

Values are n/N (%) unless stated. ARR, absolute risk reduction (difference in proportions, probiotic minus control); NNT, number needed to treat (=1/ARR); OR, odds ratio; CI, confidence interval. The prespecified responder outcome was IBS-SSS reduction ≥50 points at 8–12 weeks. ARR is expressed as percentage points; positive values favour probiotics. NNT is calculated as 1/ARR; where the ARR 95% CI crosses zero, NNT is not estimable (NE). Odds ratios and their 95% CIs are from Fisher’s exact tests on 2 × 2 contingency tables. Subgroup rows correspond to prespecified strata; no formal interaction testing is presented here. All estimates pertain to the 8- to 12-week assessment window in the propensity-matched cohort.

Other prespecified secondary outcomes showed consistent separation between groups. Pain responder rates, defined as at least 30% reduction in numeric rating scale, were 58.7% in probiotic users and 40.0% in non-users (absolute risk reduction: 18.7 percentage points, 95% CI: 5.1 to 32.2; number needed to treat: 5.3, 95% CI: 3.1 to 19.6; odds ratio: 2.1, 95% CI: 1.1 to 4.2). Adequate relief, defined as yes in at least 50% of study weeks, occurred in 54.7% versus 38.7%, respectively (absolute risk reduction: 16.0 percentage points, 95% CI: 2.7 to 29.3; number needed to treat: 6.3, 95% CI: 3.4 to 37.0; odds ratio: 1.9, 95% CI: 1.0 to 3.7) (Figures 5A,B; Table 4). Quality of life improved to a greater extent with probiotics, with a mean change in IBS-QOL of 16.0 points versus 8.0 points, corresponding to a mean difference of 8.0 points (95% CI: 3.0 to 13.0; *p* = 0.002); the distributional and effect-summary views are presented in Figures 3B,C and Table 4. The proportion exceeding the IBS-QOL minimal clinically important difference of at least 14 points was 53.3% versus 34.7% (absolute risk reduction: 18.7 percentage points, 95% CI: 5.0 to 32.3; number needed to treat: 5.3, 95% CI: 3.1 to 20.0; odds ratio: 2.2, 95% CI: 1.1 to 4.2) (Figures 5A,B; Table 4).

**Table 4:** Key secondary outcomes in the matched cohort.

Outcome	Probiotic users	Non-users	ARR (95% CI), percentage points	OR (95% CI)	NNT (95% CI)	Additional estimates
IBS-SSS MCID responder	47/75 (62.7)	33/75 (44.0)	18.7 (5.9 to 31.5)	2.2 (1.1 to 4.3)	5.3 (3.2 to 16.9)	—
Pain ≥30% responder	44/75 (58.7)	30/75 (40.0)	18.7 (5.1 to 32.2)	2.1 (1.1 to 4.2)	5.3 (3.1 to 19.6)	—
Adequate relief	41/75 (54.7)	29/75 (38.7)	16.0 (2.7 to 29.3)	1.9 (1.0 to 3.7)	6.3 (3.4 to 37.0)	—
ΔIBS-QOL (mean change, points)	16.0 (12.0)*	8.0 (12.0)*	—	—	—	MD 8.0 (95% CI 3.0 to 13.0), *P* = 0.002
QOL MCID responder (≥ + 14)	40/75 (53.3)	26/75 (34.7)	18.7 (5.0 to 32.3)	2.2 (1.1 to 4.2)	5.3 (3.1 to 20.0)	—

Values are n/N (%) unless stated. ARR, absolute risk reduction (probiotic minus control, percentage points); NNT, number needed to treat (=1/ARR); OR, odds ratio; CI, confidence interval; MD, mean difference. Responder definitions: IBS-SSS MCID, reduction ≥50 points; Pain responder, ≥30% reduction on the numeric rating scale; Adequate relief, “yes” in ≥50% of study weeks; QOL MCID, increase ≥14 points on IBS-QOL. All outcomes assessed at 8–12 weeks in the propensity-matched cohort. Proportions are shown with Wilson 95% confidence intervals; odds ratios and their 95% CIs were obtained from Fisher’s exact tests on 2 × 2 tables. Mean difference in ΔIBS-QOL was estimated using an independent-samples t-test with unequal variances (Welch). NNT is reported only when the ARR 95% CI does not cross zero; CIs were derived from the ARR interval by inversion. All analyses pertain to the 8- to 12-week assessment window in the propensity-matched cohort; no multiplicity adjustment was applied to secondary outcomes.

### 3.5 Earlier and sustained symptom improvements evident by 4–6 weeks

In the matched cohort, temporal analyses demonstrated that both groups experienced improvement in IBS symptoms from baseline, with earlier and greater reductions observed in probiotic users. Mean IBS-SSS declined from baseline in both groups, with earlier and greater improvement among probiotic users. The trajectories had already separated by 4–6 weeks and diverged further by 8–12 weeks, consistent with the primary ΔIBS-SSS finding at the prespecified endpoint window (Figure 4A; Supplementary Table S2). A similar pattern was observed for pain intensity: Mean NRS scores decreased from 6.1 at baseline to 4.8 and 4.1 at 4–6 weeks and 8–12 weeks in probiotic users, compared with 6.0, 5.4, and 4.9 in non-users (Figure 4B; Supplementary Table S2). Stool-form trajectories showed the same directional pattern: The proportion of days with Bristol types 6 or 7 fell from 49% at baseline to 39 and 34% at 4–6 weeks and 8–12 weeks in probiotic users, compared with 48, 43, and 39% in non-users; by 8–12 weeks, the absolute between-group difference reached 5 percentage points (Figure 4C; Supplementary Table S2). Consistent with these continuous outcomes, clinically meaningful symptom response became more frequent over time. IBS-SSS responder rates increased from 45.8% versus 30.1% at 4–6 weeks to 62.7% versus 44.0% at 8–12 weeks in probiotic users and non-users, respectively (Figure 4D; Supplementary Table S2). These descriptive temporal patterns indicate that probiotic-associated improvement was already apparent at the interim follow-up window and became more pronounced by 8–12 weeks.

### 3.6 Greater benefits in diarrhoea-predominant and severe baseline subgroups

In analyses stratified by IBS subtype, both IBS-D and IBS-M patients demonstrated improvements in symptom severity with probiotic use, although the magnitude was greater among those with IBS-D. At 8–12 weeks, the mean difference in ΔIBS-SSS between probiotic users and non-users was −35 points in IBS-D and −33 points in IBS-M, with both estimates statistically significant (Table 5). The proportion achieving the prespecified MCID responder threshold was 68.1% in probiotic users versus 45.7% in non-users for IBS-D, corresponding to an odds ratio of 2.6 (95% CI: 1.1 to 6.2), whereas for IBS-M the rates were 53.6 and 41.4%, respectively, with an odds ratio of 1.6 (95% CI: 0.6 to 4.3) (Figure 6). Patterns for pain reduction, adequate relief, and quality-of-life response followed the same direction, with larger absolute benefits in the IBS-D subgroup (Table 6).

**Table 5:** Mean difference in ΔIBS-SSS at 8–12 weeks by predefined strata in the matched cohort.

Stratum	Probiotic users, ΔIBS-SSS [mean (SD), *n*]	Non-users, ΔIBS-SSS [mean (SD), *n*]	MD (95% CI)	*p*-value
Overall	−95 (40), *n* = 75	−60 (40), *n* = 75	−35 (−50 to −20)	0.001
IBS-D subtype	−100 (42), *n* = 47	−65 (39), *n* = 46	−35 (−53 to −17)	0.002
IBS-M subtype	−88 (38), *n* = 28	−55 (37), *n* = 29	−33 (−54 to −12)	0.004
Baseline IBS-SSS < 300	−75 (35), *n* = 28	−50 (34), *n* = 28	−25 (−43 to −7)	0.007
Baseline IBS-SSS ≥ 300	−110 (38), *n* = 47	−68 (40), *n* = 47	−42 (−58 to −26)	<0.001

Values are mean (SD) unless otherwise indicated. ΔIBS-SSS = follow-up score at 8–12 weeks minus baseline; negative values indicate improvement. MD, mean difference (probiotic minus control); CI, confidence interval. *p*-values from Welch’s *t*-tests. All data are from the propensity score-matched analytic set (*n* = 75 per group). Numbers vary by stratum due to distribution of baseline subtype and severity. Negative MD values indicate greater improvement in the probiotic group. Estimates are based on simple two-sample comparisons within strata and are descriptive; no formal tests for interaction were conducted. Missing data were not imputed; denominators reflect patients with available 8–12-week assessments. *p*-values are provided for reference and have not been adjusted for multiple testing.

**Table 6:** Stratified summary of primary and key secondary outcomes in the matched cohort.

Stratum	Outcome	Probiotic users	Non-users	Effect estimate
Overall	ΔIBS-SSS, mean (SD), *n*	−95 (40), *n* = 75	−60 (40), *n* = 75	MD − 35 (95% CI − 50 to −20), *p* = 0.001
	MCID responder (≥50↓), n/N (%)	47/75 (62.7)	33/75 (44.0)	ARR 18.7 pp.; OR 2.2 (95% CI 1.1 to 4.3); NNT 5.3
	Pain ≥30% responder, n/N (%)	44/75 (58.7)	30/75 (40.0)	ARR 18.7 pp.; OR 2.1 (95% CI 1.1 to 4.2); NNT 5.3
	Adequate relief, n/N (%)	41/75 (54.7)	29/75 (38.7)	ARR 16.0 pp.; OR 1.9 (95% CI 1.0 to 3.7); NNT 6.3
	QOL MCID responder (≥ + 14), n/N (%)	40/75 (53.3)	26/75 (34.7)	ARR 18.7 pp.; OR 2.2 (95% CI 1.1 to 4.2); NNT 5.3
IBS-D subtype	ΔIBS-SSS, mean (SD), *n*	−100 (42), *n* = 47	−65 (39), *n* = 46	MD − 35 (95% CI − 53 to −17), P = 0.002
	MCID responder (≥50↓), n/N (%)	32/47 (68.1)	21/46 (45.7)	ARR 22.4 pp.; OR 2.6 (95% CI 1.1 to 6.2); NNT 4.5
	Pain ≥30% responder, n/N (%)	30/47 (63.8)	17/46 (37.0)	ARR 26.8 pp.; OR 3.0 (95% CI 1.3 to 7.0); NNT 3.7
	Adequate relief, n/N (%)	27/47 (57.4)	16/46 (34.8)	ARR 22.6 pp.; OR 2.5 (95% CI 1.1 to 5.9); NNT 4.4
	QOL MCID responder (≥ + 14), n/N (%)	27/47 (57.4)	15/46 (32.6)	ARR 24.8 pp.; OR 2.8 (95% CI 1.2 to 6.5); NNT 4.0
IBS-M subtype	ΔIBS-SSS, mean (SD), *n*	−88 (38), *n* = 28	−55 (37), *n* = 29	MD − 33 (95% CI − 54 to −12), *p* = 0.004
	MCID responder (≥50↓), n/N (%)	15/28 (53.6)	12/29 (41.4)	ARR 12.2 pp.; OR 1.6 (95% CI 0.6 to 4.3); NNT NE
	Pain ≥30% responder, n/N (%)	14/28 (50.0)	13/29 (44.8)	ARR 5.2 pp.; OR 1.2 (95% CI 0.4 to 3.5); NNT 19.2
	Adequate relief, n/N (%)	14/28 (50.0)	13/29 (44.8)	ARR 5.2 pp.; OR 1.2 (95% CI 0.5 to 3.4); NNT 19.2
	QOL MCID responder (≥ + 14), n/N (%)	13/28 (46.4)	11/29 (37.9)	ARR 8.5 pp.; OR 1.4 (95% CI 0.5 to 4.1); NNT 11.8
Baseline IBS-SSS < 300	ΔIBS-SSS, mean (SD), *n*	−75 (35), *n* = 28	−50 (34), *n* = 28	MD − 25 (95% CI − 43 to −7), *p* = 0.007
	MCID responder (≥50↓), n/N (%)	14/28 (50.0)	12/28 (42.9)	ARR 7.1 pp.; OR 1.3 (95% CI 0.5 to 3.7); NNT NE
	Pain ≥30% responder, n/N (%)	15/28 (53.6)	11/28 (39.3)	ARR 14.3 pp.; OR 1.8 (95% CI 0.6 to 5.2); NNT NE
	Adequate relief, n/N (%)	14/28 (50.0)	10/28 (35.7)	ARR 14.3 pp.; OR 1.8 (95% CI 0.6 to 5.3); NNT NE
	QOL MCID responder (≥ + 14), n/N (%)	13/28 (46.4)	9/28 (32.1)	ARR 14.3 pp.; OR 1.8 (95% CI 0.6 to 5.4); NNT NE
Baseline IBS-SSS ≥ 300	ΔIBS-SSS, mean (SD), *n*	−110 (38), *n* = 47	−68 (40), *n* = 47	MD − 42 (95% CI − 58 to −26), *p* < 0.001
	MCID responder (≥50↓), n/N (%)	33/47 (70.2)	21/47 (44.7)	ARR 25.8 pp.; OR 2.9 (95% CI 1.3 to 6.7); NNT 3.9
	Pain ≥30% responder, n/N (%)	29/47 (61.7)	19/47 (40.4)	ARR 21.3 pp.; OR 2.4 (95% CI 1.0 to 5.4); NNT 4.7
	Adequate relief, n/N (%)	27/47 (57.4)	19/47 (40.4)	ARR 17.0 pp.; OR 2.0 (95% CI 0.9 to 4.5); NNT 5.9
	QOL MCID responder (≥ + 14), n/N (%)	27/47 (57.4)	16/47 (34.0)	ARR 23.4 pp.; OR 2.6 (95% CI 1.1 to 6.0); NNT 4.3

Values are mean (SD) or n/N (%) as indicated. ΔIBS-SSS = IBS-SSS at 8–12 weeks minus baseline; negative values indicate improvement. MD, mean difference (probiotic minus control); ARR, absolute risk reduction (percentage points, probiotic minus control); NNT, number needed to treat (=1/ARR); OR, odds ratio; CI, confidence interval. Binary outcome ORs and CIs were obtained from Fisher’s exact tests; MD CIs and p-values from Welch’s two-sample t-tests. All estimates are derived from the propensity score-matched cohort at the 8- to 12-week assessment; denominators reflect available data within each stratum. For ΔIBS-SSS, negative MD favours probiotics. For binary outcomes, positive ARR and OR greater than 1 favour probiotics. NNT is shown only when ARR is positive and its 95% CI does not cross zero; NE denotes not estimable. These stratified analyses were prespecified and are descriptive, intended to address “who benefits more”; no formal interaction testing was performed. Missing data were not imputed. Confidence intervals for OR were obtained from Fisher’s exact tests on 2 × 2 tables; continuous outcomes used Welch’s *t*-test. Multiple testing adjustments were not applied to stratified outcomes; interpretation should consider the exploratory nature of these analyses.

When stratified by baseline symptom severity, patients with more severe symptoms (IBS-SSS ≥ 300) showed a larger treatment effect than those with moderate baseline severity. The mean difference in ΔIBS-SSS was −42 points in the severe subgroup compared with −25 points in the moderate subgroup (Table 5). The MCID responder rate reached 70.2% in probiotic users versus 44.7% in non-users with severe baseline symptoms, corresponding to an odds ratio of 2.9 (95% CI: 1.3 to 6.7), whereas the moderate-severity subgroup showed a smaller difference, 50.0% versus 42.9%, with an odds ratio of 1.3 (95% CI: 0.5 to 3.7) (Figure 6). Similar gradients were observed across pain response, adequate relief, and IBS-QOL outcomes, with more consistent and clinically meaningful benefits among patients with severe baseline symptom burden (Table 6).

### 3.7 Enhanced responses in suspected bile acid phenotype and high-adherence subsets

**Figure 7:**
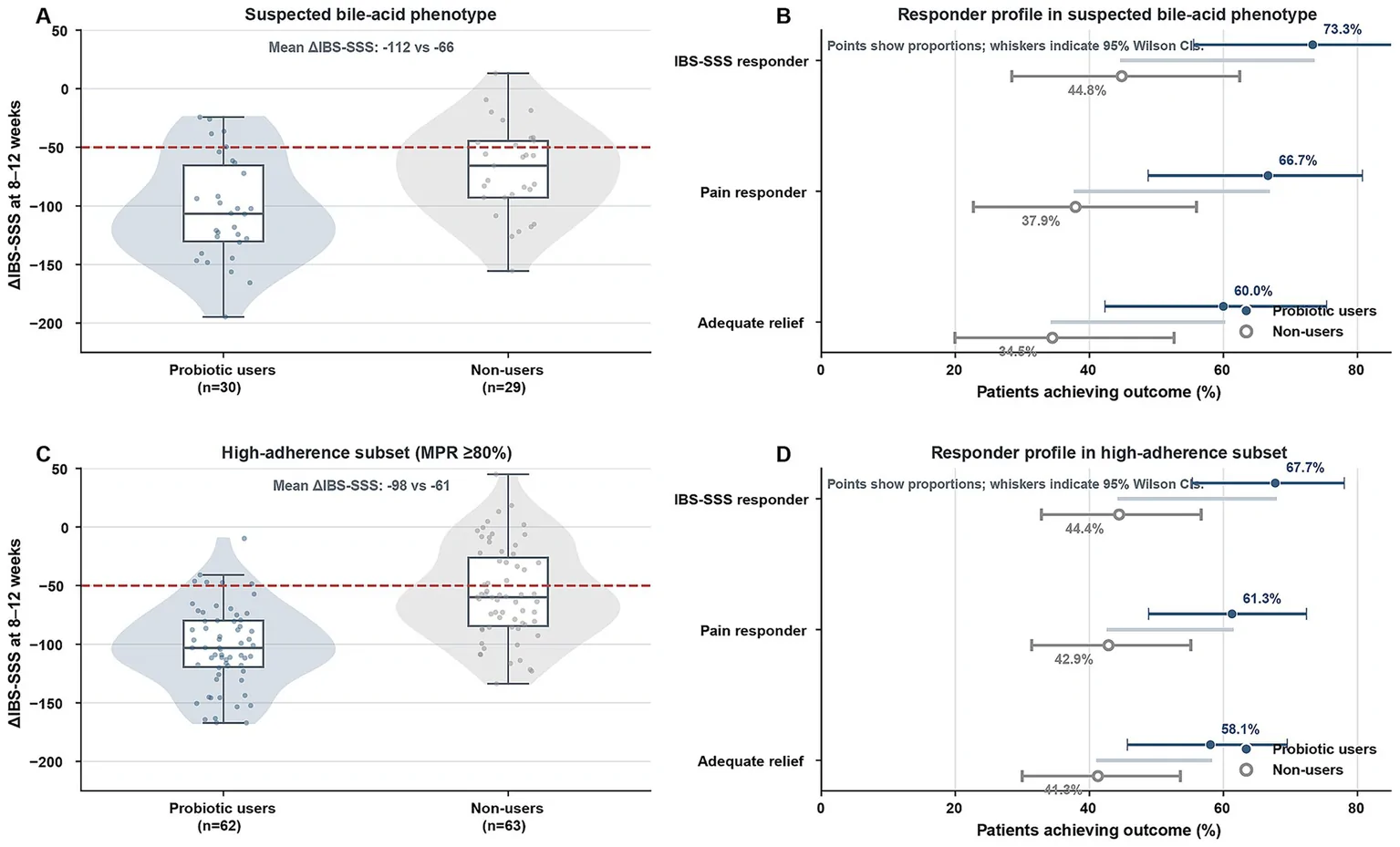
Exploratory response patterns in clinically relevant nested subsets. Panel **(A)** shows the distribution of change in IBS Symptom Severity Score (ΔIBS-SSS) from baseline to 8–12 weeks in the clinical-criteria subset with a suspected bile acid-related phenotype; negative values indicate improvement, and the dashed line denotes the minimal clinically important difference threshold of −50 points. Panel **(B)** shows the corresponding responder profiles with 95% Wilson confidence intervals for IBS-SSS response, pain response, and adequate relief in the same subset. Panels **(C,D)** present the analogous analyses for the high-adherence subset, defined by a medication possession ratio of at least 80%. All nested subset analyses are descriptive, derived from the matched cohort, and intended to illustrate clinically relevant heterogeneity without formal interaction testing. The suspected bile acid-related phenotype was defined using prespecified clinical criteria rather than confirmatory bile acid biomarker testing; therefore, these findings should be considered hypothesis-generating.

In exploratory nested analyses restricted to the clinical-criteria subset with a suspected bile acid-related phenotype, as defined in the Methods, probiotic users showed a more favourable pattern of symptom improvement than non-users. Mean ΔIBS-SSS values were −112 versus −66, and the distribution at 8–12 weeks was shifted further beyond the prespecified minimal clinically important difference threshold of −50 points in probiotic users, whereas non-users showed more modest improvement (Figure 7A). Responder profiles were directionally concordant, with higher observed proportions of IBS-SSS response, pain response, and adequate relief in probiotic users than in non-users (Figure 7B).

A similar pattern was observed in the high-adherence subset, defined by a medication possession ratio of at least 80%. In this subset, probiotic users again showed greater reductions in IBS-SSS over 8–12 weeks than non-users, with mean ΔIBS-SSS values of −98 versus −61 and a distribution that more clearly exceeded the minimal clinically important difference threshold (Figure 7C). Responder outcomes were also directionally consistent with the primary analysis, with higher observed proportions of IBS-SSS response, pain response, and adequate relief in probiotic users than in non-users (Figure 7D). Together, these descriptive nested analyses suggest that higher observed response rates were concentrated among patients with bile acid suggestive clinical features and among patients with better adherence. These findings should be interpreted as hypothesis-generating because the bile acid-related phenotype was clinically suspected rather than biomarker-confirmed, and adherence was measured after treatment initiation (Figure 7).

### 3.8 Comparable safety profile with lower need for treatment escalation

Adverse events were infrequent and generally mild in both groups (Table 7). Any adverse event was reported in 12 of 75 probiotic users (16.0%) and 10 of 75 non-users (13.3%), with no significant between-group difference. The most common events were bloating and transient worsening of abdominal pain, observed in 12.0% versus 8.0 and 6.7% versus 9.3% of patients in the probiotic and control groups, respectively. Discontinuation due to adverse events was rare, occurring in three probiotic users (4.0%) and two non-users (2.7%). Only one serious adverse event was recorded, in the control group, and no probiotic-related serious events were identified. Treatment escalation within 8–12 weeks was less frequent among probiotic users than non-users, occurring in 18 of 75 patients (24.0%) versus 30 of 75 patients (40.0%), corresponding to an absolute reduction of 16.0 percentage points (95% CI: 1.1 to 30.0) and an odds ratio of 0.47 (95% CI: 0.24 to 0.94; *p* = 0.03) (Figures 5A,B). The most frequent escalation measure was initiation of add-on medication, required in 20.0% of probiotic users and 33.3% of non-users, followed by emergency department visits (5.3% versus 10.7%) and hospitalisations for IBS-related complaints (1.3% versus 4.0%). The frequency of treatment escalation components, including initiation of add-on medication, emergency department visits, and hospitalisations, was numerically lower in probiotic users (Table 7).

**Table 7:** Safety events and treatment escalation within 8–12 weeks in the matched cohort.

Outcome	Probiotic users	Non-users	RD, pp (95% CI)	OR (95% CI)	*p*-value
Any adverse event	12/75 (16.0)	10/75 (13.3)	+2.7 (−8.2 to +13.6)	1.24 (0.52 to 2.98)	0.65
Bloating	9/75 (12.0)	6/75 (8.0)	+4.0 (−6.3 to +14.3)	1.57 (0.56 to 4.47)	0.42
Abdominal pain worsening	5/75 (6.7)	7/75 (9.3)	−2.6 (−11.2 to +6.0)	0.70 (0.21 to 2.29)	0.55
Discontinuation due to AE	3/75 (4.0)	2/75 (2.7)	+1.3 (−5.2 to +7.8)	1.50 (0.24 to 9.22)	0.68
Serious adverse event (SAE)	0/75 (0.0)	1/75 (1.3)	−1.3 (−3.9 to +1.3)	NE	1.00
Treatment escalation (any)^a^	18/75 (24.0)	30/75 (40.0)	−16.0 (−30.1 to −1.9)	0.47 (0.24 to 0.94)	0.03
Add-on medication^b^	15/75 (20.0)	25/75 (33.3)	−13.3 (−26.6 to +0.0)	0.50 (0.24 to 1.03)	0.08
Emergency department visit	4/75 (5.3)	8/75 (10.7)	−5.4 (−13.6 to +2.8)	0.47 (0.14 to 1.61)	0.22
Hospitalisation (IBS-related)	1/75 (1.3)	3/75 (4.0)	−2.7 (−8.1 to +2.7)	0.32 (0.03 to 3.18)	0.62

Values are n/N (%) unless otherwise stated. RD, risk difference (percentage points; probiotic minus control); OR, odds ratio; CI, confidence interval. *p*-values are from *χ*^2^ tests or Fisher’s exact tests as appropriate. Safety and escalation events were assessed within the 8- to 12-week window in the propensity score-matched cohort. Adverse events (AEs) were abstracted from electronic health records and follow-up notes; denominators reflect patients with available safety assessments in the 8- to 12-week window. Risk differences are shown as percentage points with Newcombe-type confidence intervals; odds ratios and their 95% CIs were derived from Fisher’s exact tests when expected counts were small. NE denotes not estimable due to zero cells.

a Composite “treatment escalation” includes any of the following within 8–12 weeks: initiation of add-on pharmacotherapy, emergency department visit, or hospitalisation for IBS-related complaints.

b Add-on pharmacotherapy includes initiation of antispasmodics, loperamide, rifaximin, bile acid sequestrants, or low-dose tricyclic antidepressants as documented in charts. No adjustment for multiplicity was applied to safety or escalation endpoints; the results are interpreted descriptively in the context of the matched cohort.

## 4 Discussion

The present single-centre, propensity score-matched, real-world cohort provides evidence that initiation of a multi-strain probiotic in routine Chinese hospital care was associated with clinically meaningful improvements in symptoms and quality of life among adults with Rome IV irritable bowel syndrome of diarrhoea-predominant or mixed subtype (38, 42). In the matched set of 150 patients, the mean reduction in IBS Symptom Severity Score over 8 to 12 weeks was larger with probiotics than with contemporary standard care, the between-group mean difference was approximately 35 points with confidence limits that exclude a trivial effect, and separation of trajectories was already apparent by 4 to 6 weeks and increased thereafter (35). Proportions meeting prespecified responder definitions also favoured probiotics for the symptom severity threshold of at least 50 points, for a reduction in pain intensity of at least 30%, for adequate relief in at least half of the observed weeks, and for the quality of life minimal clinically important difference (41). These gains occurred without an excess of adverse events, while escalation of treatment within 8 to 12 weeks was less frequent in probiotic users (43). Together, these findings indicate that the signal for benefit is coherent across continuous and categorical endpoints, appears early, reaches a magnitude that is likely perceptible to patients, and coincides with a stable safety profile in routine care.

Interpretation of effect sizes against minimal clinically important difference thresholds is central for clinical decision-making. The observed mean difference in IBS Symptom Severity Score was of a size that would be expected to translate into noticeable relief for many patients (22, 44). The distributional view from the violin–box plots shows that a larger share of probiotic users exceeded the 50-point threshold, while the longitudinal trajectories showed progressive group separation over follow-up (22). The time course matters operationally for clinics that schedule follow-up visits every 4 to 6 weeks. In such cycles, an early review can be used to assess whether a patient is on a trajectory to reach clinically important improvement and to decide whether to maintain, combine, or switch therapy. The convergence of responder analyses with the minimal clinically important difference anchored continuous outcomes supports the internal consistency of the signal (45). Improvements in disease specific quality of life further suggest that symptom gains were not merely statistical artefacts but translated into domains that patients value (46, 47).

Heterogeneity of response is a frequent reason for modest average effects in functional disorders. In prespecified analyses, we observed larger associations in diarrhoea-predominant disease and in patients with severe baseline symptom burden (48, 49). These are clinically recognisable phenotypes that often present with frequent loose stools, urgency, and episodic flares that impair daily activities. The pattern is biologically plausible. Combinations of lactic acid bacteria and bifidobacteria may modulate microbial metabolites, reinforce epithelial barrier function, and attenuate low-grade mucosal immune activation. Bile salt hydrolase activity present in several probiotic taxa can deconjugate bile acids, potentially reducing the pool of colonic bile acids with secretory and motor effects (50, 51). Such mechanisms align with the greater associations seen in diarrhoea-predominant disease and with the descriptive signal in the nested subset with features suggestive of bile acid involvement. The high adherence subset also showed higher responder proportions. Adherence is a determinant of observed effectiveness for any orally administered therapy, and the pattern observed here is consistent with pharmacologic common sense (52). Because adherence is measured after treatment initiation, analyses were descriptive and not used to draw causal inferences. However, they provide pragmatic reassurance that the benefits observed in the main matched cohort are unlikely to be confined to sporadic users.

Comparison with existing literature is complex because probiotic interventions vary widely by species, strain, dose, and formulation. Previous trials and meta-analyses have reported heterogeneous results across subtypes and outcomes (16, 53, 54). Heterogeneity likely reflects differences in case mix, phenotype distribution, adherence support, and background co-medication. The present study contributes by examining a pragmatic setting in China where antispasmodics, loperamide, rifaximin, and bile acid sequestrants are frequently used in combination (19). Against this background, probiotics were associated with additional symptom and quality-of-life gains and with fewer treatment intensifications (55). These findings can be read as complementary to guideline statements that are cautious about recommending probiotics routinely for all patients with irritable bowel syndrome. Our results suggest that in clinical phenotypes where diarrhoeal mechanisms and symptom burden are prominent, and when adherence can be sustained, a multi-strain probiotic can be a reasonable component of care. At the same time, product selection, dose, and treatment duration were not standardised by protocol but reflected routine prescribing practice. Batch-level strain identifiers and batch-specific viable counts were not routinely captured in the electronic record. Therefore, the study should be interpreted as a real-world comparative effectiveness analysis of routinely prescribed multi-strain probiotic preparations, not as evidence for a specific strain, product, dose, or causal dose–response relationship.

Evidence from Chinese populations is broadly consistent with this pragmatic signal but remains methodologically uneven. Earlier, Chinese clinical reports and meta-analyses suggested that commonly used probiotic or microecological preparations may improve diarrhoeal symptoms and global IBS outcomes in routine practice, and a recent randomised study from Western China also reported symptom improvement when probiotics were combined with dietary intervention (18, 19, 41). However, much of the Chinese literature has been based on small single-centre studies, short follow-up, heterogeneous formulations, or non-standardised outcome definitions, and few studies have used a new-user design, symmetrical landmarking, propensity score balancing, MCID-anchored patient-reported outcomes, and treatment-escalation endpoints (19, 56). Compared with these prior Chinese data, the present study adds a hospital-based real-world comparison using electronic medical record and dispensing data, explicit follow-up windows, and clinically interpretable response thresholds. Direct comparison should nevertheless be cautious because probiotic products, background co-medications, dietary practice, documentation quality, and follow-up pathways vary across Chinese hospitals (18, 19).

Safety is a practical concern for any adjunctive therapy that may be used for weeks to months. In this study, adverse events were infrequent, mostly mild, and similar between groups, which is consistent with long-standing experience of the component organisms in food and supplement use (16, 53). The reduction in treatment escalation is notable because it aggregates clinically relevant consequences of persistent symptoms, including the need to add further pharmacotherapy and unscheduled care such as emergency department visits or hospitalisation (35, 57). While the composite was driven mainly by medication intensification, and while causal attribution cannot be established in a retrospective design, the pattern suggests that symptom control with probiotics may lessen downstream resource use (58). This observation warrants formal confirmation in prospective economic analyses because even small absolute reductions in unscheduled visits can be meaningful in health systems with large patient volumes.

Several features of the design strengthen inference. The study used a new-user framework with a symmetrical 14-day landmark applied to both groups to mitigate immortal time bias (59). The propensity score model included demographic, clinical, psychometric, treatment, utilisation, and calendar time variables, and matching achieved very good covariate balance with standardised mean differences within prespecified thresholds (60). The estimand in the matched analysis is the average treatment effect on the treated, which is the effect among patients for whom clinicians considered probiotics in routine practice (61). Outcomes and follow-up windows were prespecified. The primary endpoint was anchored to a recognised minimal clinically important difference (62). Sensitivity analyses using non-parametric estimators and matched pair robust inferences did not materially change conclusions. Secondary and stratified outcomes were exploratory, and false discovery rate results presented in the Supplementary Table S6 did not alter key inferences. Missing data sensitivity analyses that modelled potentially informative follow-up using inverse probability weights and pattern-mixture scenarios supported the direction of effects (63). These features, taken together, provide a coherent framework that is proportionate to the retrospective design and consistent with standards for real-world effectiveness studies.

Important limitations should be considered when interpreting the results. Residual confounding cannot be excluded despite extensive adjustment and matching (64). IBS is also characterised by substantial expectation and placebo-related responses, with meta-analytic evidence showing considerable placebo response rates in IBS trials (65). Because this was a retrospective real-world comparison rather than a blinded placebo-controlled trial, probiotic users received an additional oral preparation whereas non-users did not receive a matched placebo capsule or powder. Subjective outcomes such as IBS-SSS, pain NRS, adequate relief, and IBS-QOL may therefore have been influenced by treatment expectation, adherence behaviour, and clinician–patient interaction (65). Accordingly, the magnitude of benefit may be overestimated relative to a blinded efficacy trial, and the findings should be interpreted as real-world associations rather than proof of a probiotic-specific biological effect. Physician preference, patient diet including adherence to low fermentable oligo-di-monosaccharide and polyol guidance, self-management behaviours, and prior response to antibiotics or bile acid sequestrants were not consistently captured in the electronic record (66). Regression to the mean is always a concern when changes from baseline are analysed, although matching on baseline symptom severity and use of distribution free estimators help to mitigate this issue (67). The study relied on routine assessments and documentation. Weekly adequate relief entries and the exact timing of pain ratings may vary across visits and clinicians, and misclassification in either direction would tend to bias estimates towards the null (68). Serious adverse events were rare, and the study was not powered to detect small safety differences (35). The composite of treatment escalation was not guided by a standardised step up protocol and thus reflects both clinical need and clinician preference. Product composition, batch-level strain identifiers, dose, and treatment duration were not standardised across patients; therefore, the study cannot adjudicate strain-specific or dose-specific efficacy. Exploratory analyses based on adherence or treatment duration should be interpreted as descriptive because exposure intensity was determined after treatment initiation and may reflect symptom severity, physician preference, and patient adherence. The single-centre setting, the focus on diarrhoea-predominant and mixed subtypes, and the 8- to 12-week follow-up window limit generalisability to constipation predominant disease, to longer term maintenance, and to other healthcare systems. Finally, adherence is a post-treatment variable and the nested adherence analysis is descriptive (66). For these reasons, the findings should be interpreted as associations rather than proof of causality.

Notwithstanding these limitations, the consistency of results across multiple endpoints, the early emergence of separation, and the alignment with plausible mechanisms provide a coherent narrative for clinical use. For day to day practice in Chinese hospitals, a time limited trial of a multi-strain probiotic can be considered for patients with diarrhoea-predominant or severe symptoms, especially when clinical features suggest bile acid involvement (19, 56). The practical steps are straightforward. Initiate therapy, arrange review at 4 to 6 weeks, and use minimal clinically important difference anchored thresholds alongside patient reported adequate relief to decide whether to continue or adjust treatment (20). Attention to adherence is essential because the descriptive analyses indicate larger observed gains when medication possession ratio exceeds 80%. In non-responders, particularly those with severe ongoing diarrhoea, clinicians should evaluate diet, consider bile acid sequestrants or short courses of rifaximin as appropriate, and address psychological comorbidity when present (69). The safety profile observed here should reassure clinicians who worry about adding pills in already complex regimens, although vigilance for bloating and transient discomfort remains sensible.

The present findings also suggest specific directions for future research. Prospective enriched studies are needed to test the strategy in patients most likely to benefit (70). Enrichment could be based on clinical markers of bile acid involvement, on baseline severity, and on adherence readiness (34). Pragmatic randomised designs that compare probiotics to bile acid sequestrants, rifaximin, or to a stepped strategy that sequences these options could quantify incremental benefit over usual care. Low burden biomarker panels that include faecal bile acids, serum 7 alpha hydroxy 4 cholesten 3 one, and fibroblast growth factor 19 may help to characterise phenotypes and to understand mechanisms without imposing onerous sampling (71). Embedding adherence support interventions and measuring their effect on effectiveness would address a fundamental determinant of outcomes. Cost effectiveness analyses that incorporate treatment escalation and unscheduled care should be included because these endpoints were sensitive to probiotic use in the present study (70). Longer follow-up will be necessary to determine whether benefits are durable, whether continuous or intermittent dosing is preferable, and whether withdrawal leads to relapse (72). Multi-centre collaborations would help to assess generalisability across dietary patterns, prescribing cultures, and health systems (73).

It is also important to place these results within the policy environment and guideline landscape. Many international guidelines remain cautious about routine probiotic use in irritable bowel syndrome, in part because strain composition and trial quality vary widely and because negative studies exist alongside positive ones (74, 75). Real-world evidence from a Chinese hospital context where co-medications are commonly used and where adherence can be supported adds a different form of data that is complementary rather than contradictory (16, 20). Such evidence does not replace randomised trials but can guide the design of future pragmatic studies and can inform clinicians who must make decisions for patients who are not captured by narrow eligibility criteria (76). The present study targets the population for whom probiotics are actually considered and used, which is the relevant population for practice.

## 5 Conclusion

In a propensity-matched cohort of adults with diarrhoea-predominant and mixed irritable bowel syndrome in routine Chinese hospital care, initiation of a multi-strain probiotic was associated with greater reductions in symptom severity, higher responder proportions across prespecified endpoints, meaningful improvements in disease specific quality of life, and a lower need for treatment escalation over 8 to 12 weeks, with adverse events that were infrequent and mild. The signal appears stronger in clinically recognisable phenotypes and when adherence is high. These findings support considering a targeted and time-limited trial of routinely prescribed multi-strain probiotic preparations with structured reassessment, while confirmation of efficacy and separation of biological effects from expectation effects will require blinded placebo-controlled or pragmatic randomised studies. They also define clear priorities for prospective enriched pragmatic studies that can validate the magnitude and durability of benefit, identify the patients most likely to respond, and quantify the economic consequences of reduced escalation and unscheduled care.

## Data Availability

The raw data supporting the conclusions of this article will be made available by the authors, without undue reservation.
