# Supplementary material for: Initiating multi-strain probiotics is associated with clinically meaningful symptom relief, better quality of life, and fewer treatment escalations in IBS-D/IBS-M: a Chinese real-world propensity-matched cohort study
Source: Front Med (Lausanne). 2026 Aug 18;13:1900112. doi: 10.3389/fmed.2026.1900112 (PMC13529527; doi:10.3389/fmed.2026.1900112)
Supplement: Supplementary file 1 [file Table_1.DOCX]

**Supplementary Table S1. Baseline characteristics before propensity score matching**

| Variable | Probiotic users (n=122) | Non-users (n=165) | SMD (before matching) |
| --- | --- | --- | --- |
| **Demographics** |  |  |  |
| Age, years | 41.8 (12.1) | 43.7 (12.4) | 0.15 |
| Female, n (%) | 72 (59.0) | 92 (55.8) | 0.06 |
| BMI, kg/m² | 22.9 (3.1) | 22.7 (3.0) | 0.06 |
| **Disease profile** |  |  |  |
| IBS subtype—D, n (%) | 83 (68.0) | 89 (53.9) | 0.29 |
| IBS subtype—M, n (%) | 39 (32.0) | 76 (46.1) | 0.29 |
| Disease duration, years, median (IQR) | 3.4 (1.3–6.5) | 2.9 (1.1–5.6) | 0.12 |
| IBS-SSS (0–500) | 334 (66) | 306 (63) | 0.43 |
| Pain NRS (0–10) | 6.3 (1.4) | 5.8 (1.5) | 0.34 |
| IBS-QOL (0–100) | 52.1 (12.6) | 57.2 (12.1) | 0.41 |
| **Bowel habit** |  |  |  |
| Bristol 6/7 days, % | 52.4 (15.2) | 46.9 (14.5) | 0.37 |
| Bowel frequency, stools/day | 2.8 (1.1) | 2.4 (1.0) | 0.38 |
| **Comorbidity & prior utilisation** |  |  |  |
| Charlson comorbidity index | 0.8 (0.9) | 0.7 (0.8) | 0.12 |
| Anxiety/depression recorded, n (%) | 34 (27.9) | 35 (21.2) | 0.16 |
| Prior ED visit or hospitalisation, n (%) | 20 (16.4) | 20 (12.1) | 0.12 |
| **Concomitant medications (baseline ±14 days)** |  |  |  |
| Loperamide (anti-diarrhoeal), n (%) | 39 (32.0) | 30 (18.2) | 0.33 |
| Antispasmodics, n (%) | 50 (41.0) | 46 (27.9) | 0.27 |
| Bile-acid sequestrants, n (%) | 7 (5.7) | 4 (2.4) | 0.19 |
| Rifaximin, n (%) | 12 (9.8) | 8 (4.8) | 0.19 |
| Proton-pump inhibitors, n (%) | 36 (29.5) | 39 (23.6) | 0.13 |
| TCAs/SSRIs, n (%) | 18 (14.8) | 18 (10.9) | 0.12 |
| **Calendar time at index** |  |  |  |
| Quarter 1, n (%) | 37 (30.3) | 34 (20.6) | 0.22 |
| Quarter 2, n (%) | 27 (22.1) | 43 (26.1) | 0.09 |
| Quarter 3, n (%) | 29 (23.8) | 48 (29.1) | 0.11 |
| Quarter 4, n (%) | 29 (23.8) | 40 (24.2) | 0.01 |

Values are mean (SD) or n (%) unless stated. SMD, standardized mean difference; IBS-SSS, Irritable Bowel Syndrome–Symptom Severity Score (0–500); IBS-QOL, IBS–Quality of Life (0–100); NRS, numeric rating scale; BMI, body-mass index. Predefined threshold for meaningful imbalance: |SMD| ≥0.10.Baseline is defined as the index date (first probiotic prescription for users; a calendar-matched index date for non-users). Concomitant medications were captured within ±14 days of baseline. Bristol 6/7 days (%) was calculated among abnormal bowel movement days per Rome IV guidance. Disease duration is presented as median (IQR); SMD for skewed variables was approximated using pooled-variance or rank-based standardisation. No hypothesis testing is performed for baseline comparisons; effect estimates in subsequent analyses account for imbalances via propensity score matching. Missingness across covariates was low (≤3% for any single variable) and did not differ materially between groups; variables with any missing values were included in the propensity model with appropriate handling. The observed pre-match imbalances (e.g., higher IBS-SSS, greater IBS-D proportion, and more frequent anti-diarrhoeal/antispasmodic use among probiotic users) motivated propensity score matching; matched characteristics are presented in Table 1 of the main text.
