# Supplementary material for: Initiating multi-strain probiotics is associated with clinically meaningful symptom relief, better quality of life, and fewer treatment escalations in IBS-D/IBS-M: a Chinese real-world propensity-matched cohort study
Source: Front Med (Lausanne). 2026 Aug 18;13:1900112. doi: 10.3389/fmed.2026.1900112 (PMC13529527; doi:10.3389/fmed.2026.1900112)
Supplement: Supplementary file 2 [file Table_2.docx]

**Supplementary Table S2. Longitudinal outcomes at baseline, 4–6 weeks, and 8–12 weeks in the matched cohort**

| Outcome | Timepoint | Probiotic users | Non-users | MD (95% CI) | P value |
| --- | --- | --- | --- | --- | --- |
| **IBS-SSS** | Baseline | 302 (68) | 299 (70) | 3 (−21 to 27) | 0.79 |
|  | 4–6 wk | 226 (65) | 258 (66) | −32 (−55 to −9) | 0.006 |
|  | 8–12 wk | 207 (61) | 239 (64) | −32 (−54 to −10) | 0.004 |
| **Pain NRS (0–10)** | Baseline | 6.1 (1.3) | 6.0 (1.4) | 0.1 (−0.4 to 0.6) | 0.71 |
|  | 4–6 wk | 4.8 (1.5) | 5.4 (1.5) | −0.6 (−1.1 to −0.1) | 0.02 |
|  | 8–12 wk | 4.1 (1.4) | 4.9 (1.5) | −0.8 (−1.3 to −0.3) | 0.002 |
| **Bristol stool type 6/7 days (% of days)** | Baseline | 49 (15) | 48 (15) | 1 (−5 to 7) | 0.74 |
|  | 4–6 wk | 39 (14) | 43 (14) | −4 (−9 to 1) | 0.11 |
|  | 8–12 wk | 34 (13) | 39 (14) | −5 (−10 to 0) | 0.06 |
| **IBS-QOL** | Baseline | 65 (17) | 64 (16) | 1 (−5 to 7) | 0.65 |
|  | 4–6 wk | 74 (18) | 69 (17) | 5 (0 to 10) | 0.05 |
|  | 8–12 wk | 81 (17) | 72 (18) | 9 (3 to 15) | 0.004 |
| **Responder (IBS-SSS ≥50 reduction), n/N (%)** | 4–6 wk | 33/72 (45.8) | 22/73 (30.1) | +15.7% (2.0 to 29.4) | 0.02 |
|  | 8–12 wk | 47/75 (62.7) | 33/75 (44.0) | +18.7% (5.9 to 31.5) | 0.004 |

Values are mean (SD) unless otherwise indicated. MD, mean difference (probiotic minus control); CI, confidence interval; P, two-sample t test for continuous outcomes and χ² test for categorical outcomes. Negative MD denotes improvement in favour of probiotics for IBS-SSS and pain NRS; positive MD denotes improvement for IBS-QOL. All values are presented for the propensity score–matched analytic cohort (n=75 per group at baseline; n varies by timepoint due to follow-up completeness).Continuous outcomes are expressed as mean (SD); categorical outcomes are given as n/N (%).MD and CI for continuous variables were estimated using independent-sample t tests; differences in proportions were assessed with χ² tests. Missing data were not imputed; denominators reflect the number of patients with available assessments at each timepoint. All analyses are descriptive; no adjustment for multiplicity was applied.
