# Supplementary material for: Initiating multi-strain probiotics is associated with clinically meaningful symptom relief, better quality of life, and fewer treatment escalations in IBS-D/IBS-M: a Chinese real-world propensity-matched cohort study
Source: Front Med (Lausanne). 2026 Aug 18;13:1900112. doi: 10.3389/fmed.2026.1900112 (PMC13529527; doi:10.3389/fmed.2026.1900112)
Supplement: Supplementary file 3 [file Table_3.DOCX]

**Supplementary Table S3. Probiotic exposure characteristics among new users**

| **Exposure characteristic** | **Full probiotic new-user set (n=122)** | **Matched probiotic users (n=75)** |
| --- | --- | --- |
| Electronic prescription and pharmacy dispensing records available, n (%) | 122 (100.0) | 75 (100.0) |
| Product name/type, dosage strength, prescribed dose, and administration frequency documented, n (%) | 122 (100.0) | 75 (100.0) |
| Formulation-level microbial composition documented from hospital formulary/prescribing information, n (%) | 122 (100.0) | 75 (100.0) |
| Batch-level strain identifier or batch-specific viable count documented in EMR, n (%) | 0 (0.0) | 0 (0.0) |
| Oral capsule/tablet preparation, n (%) | 110 (90.2) | 68 (90.7) |
| Oral powder/granule/sachet preparation, n (%) | 12 (9.8) | 7 (9.3) |
| Bifidobacterium/Lactobacillus/Enterococcus-containing multi-strain preparation, n (%) | 62 (50.8) | 39 (52.0) |
| Bifidobacterium/Lactobacillus/Enterococcus/Bacillus-containing multi-strain preparation, n (%) | 36 (29.5) | 22 (29.3) |
| Clostridium butyricum-containing combined preparation, n (%) | 24 (19.7) | 14 (18.7) |
| Prescribed according to labelled standard adult dosing schedule, n (%) | 115 (94.3) | 71 (94.7) |
| Administration frequency: twice daily, n (%) | 43 (35.2) | 26 (34.7) |
| Administration frequency: three times daily, n (%) | 79 (64.8) | 49 (65.3) |
| Initial prescription duration, days, median (IQR) | 14 (14–28) | 14 (14–28) |
| Total documented days supplied through 8–12 weeks, median (IQR) | 56 (42–70) | 56 (42–70) |
| Medication possession ratio, %, median (IQR) | 84 (73–94) | 86 (76–96) |
| Medication possession ratio ≥80%, n (%) | 95 (77.9) | 62 (82.7) |

**Note:** Values are n (%) or median (IQR). Product type, dosage strength, prescribed dose, administration frequency, initial prescription duration, repeated dispensing, and total documented days supplied were abstracted from electronic prescription and pharmacy dispensing records. Formulation-level microbial composition was recorded according to hospital formulary information and prescribing instructions. Batch-level strain identifiers and batch-specific viable counts were not routinely captured in the electronic medical record; therefore, strain-specific analyses were not performed. Medication possession ratio was calculated as documented days supplied divided by the number of days between probiotic initiation and the 8–12-week outcome assessment, capped at 100%. Analyses by adherence or treatment duration were interpreted as exploratory exposure-intensity analyses rather than causal dose-response analyses.
