# Supplementary material for: Initiating multi-strain probiotics is associated with clinically meaningful symptom relief, better quality of life, and fewer treatment escalations in IBS-D/IBS-M: a Chinese real-world propensity-matched cohort study
Source: Front Med (Lausanne). 2026 Aug 18;13:1900112. doi: 10.3389/fmed.2026.1900112 (PMC13529527; doi:10.3389/fmed.2026.1900112)
Supplement: Supplementary file 4 [file Table_4.DOCX]

**Supplementary Table S4. Component comorbidities used to calculate the Charlson comorbidity index**

| **Variable** | **Full probiotic new-user set (n=122)** | **Full non-user set (n=165)** | **Matched probiotic users (n=75)** | **Matched non-users (n=75)** |
| --- | --- | --- | --- | --- |
| Charlson comorbidity index, mean (SD) | 0.8 (0.9) | 0.7 (0.8) | 0.7 (0.8) | 0.7 (0.8) |
| Charlson score = 0, n (%) | 74 (60.7) | 102 (61.8) | 42 (56.0) | 43 (57.3) |
| Charlson score = 1, n (%) | 31 (25.4) | 41 (24.8) | 24 (32.0) | 23 (30.7) |
| Charlson score ≥2, n (%) | 17 (13.9) | 22 (13.3) | 9 (12.0) | 9 (12.0) |
| Diabetes without end-organ damage, n (%) | 18 (14.8) | 21 (12.7) | 10 (13.3) | 9 (12.0) |
| Chronic pulmonary disease, n (%) | 12 (9.8) | 14 (8.5) | 7 (9.3) | 6 (8.0) |
| Mild liver disease, n (%) | 15 (12.3) | 18 (10.9) | 8 (10.7) | 8 (10.7) |
| Peptic ulcer disease, n (%) | 13 (10.7) | 17 (10.3) | 8 (10.7) | 7 (9.3) |
| Cardiovascular disease, n (%) | 6 (4.9) | 8 (4.8) | 4 (5.3) | 4 (5.3) |
| Cerebrovascular disease, n (%) | 5 (4.1) | 6 (3.6) | 3 (4.0) | 3 (4.0) |
| Chronic kidney disease, n (%) | 4 (3.3) | 5 (3.0) | 2 (2.7) | 2 (2.7) |
| Malignancy history, n (%) | 3 (2.5) | 3 (1.8) | 2 (2.7) | 1 (1.3) |
| Other Charlson-defined conditions, n (%) | 5 (4.1) | 6 (3.6) | 3 (4.0) | 3 (4.0) |

**Note:** Values are mean (SD) or n (%). Component comorbidities were abstracted from documented medical history and diagnosis codes recorded before or at the index visit. The Charlson comorbidity index was calculated using conventional Charlson weights. Age-adjusted Charlson scores were not used because age was entered separately as a prespecified covariate in the propensity score model. Component conditions are not mutually exclusive because some patients had more than one Charlson-defined condition. Low-frequency Charlson components were grouped as “other Charlson-defined conditions.”
