# Supplementary material for: Initiating multi-strain probiotics is associated with clinically meaningful symptom relief, better quality of life, and fewer treatment escalations in IBS-D/IBS-M: a Chinese real-world propensity-matched cohort study
Source: Front Med (Lausanne). 2026 Aug 18;13:1900112. doi: 10.3389/fmed.2026.1900112 (PMC13529527; doi:10.3389/fmed.2026.1900112)
Supplement: Supplementary file 5 [file Table_5.DOCX]

**Supplementary Table S5. Missing-data pattern and robustness checks for the primary outcome**

| **Item** | **Value / approach** |
| --- | --- |
| Patients screened during the study period | 482 |
| Excluded because of missing baseline or 8–12-week outcome data | 84/482 (17.4%) |
| Final analytic cohort after prespecified exclusions | 287 |
| Propensity score–matched cohort for primary analysis | 150 patients, 75 per group |
| Missingness in baseline covariates used for propensity score estimation | Low; no single variable had >3% missingness |
| Handling of missing baseline covariates | Median imputation for continuous variables; missing-category indicators for categorical variables |
| Primary analysis population | Complete-case matched cohort with available baseline and 8–12-week IBS-SSS |
| Primary complete-case result | MD −35 points, 95% CI −50 to −20 |
| Additional robustness checks | Non-parametric analysis, matched-pair robust inference, inverse probability of censoring weighting, and delta-adjusted pattern-mixture scenarios |
| Overall interpretation of robustness checks | Estimates were directionally consistent with the primary analysis; conservative missing-data assumptions attenuated the effect size but did not reverse the direction of association |

**Note:** IBS-SSS, Irritable Bowel Syndrome–Symptom Severity Score; MD, mean difference. Missing baseline or 8–12-week IBS-SSS data were part of the prespecified exclusion criteria. Missingness mainly reflected the absence of a documented IBS-SSS assessment within the routine follow-up window, rather than missing exposure information. Because this was a retrospective real-world study using routine clinical records, missing-data sensitivity analyses were used to evaluate robustness rather than to replace the prespecified complete-case matched analysis.
