# Supplementary material for: Initiating multi-strain probiotics is associated with clinically meaningful symptom relief, better quality of life, and fewer treatment escalations in IBS-D/IBS-M: a Chinese real-world propensity-matched cohort study
Source: Front Med (Lausanne). 2026 Aug 18;13:1900112. doi: 10.3389/fmed.2026.1900112 (PMC13529527; doi:10.3389/fmed.2026.1900112)
Supplement: Supplementary file 6 [file Table_6.docx]

**Supplementary Table S6. Benjamini–Hochberg false discovery rate-adjusted results for exploratory secondary and stratified outcomes**

| **Analysis population / stratum** | **Outcome** | **Outcome family** | **Nominal test** | **Raw P value** | **BH-adjusted P value** | **Nominal P < 0.05** | **FDR q < 0.05** |
| --- | --- | --- | --- | --- | --- | --- | --- |
| Overall matched cohort | IBS-SSS MCID responder, ≥50-point reduction | Secondary effectiveness | χ² test | 0.022 | 0.049 | Yes | Yes |
| Overall matched cohort | Pain ≥30% responder | Secondary effectiveness | χ² test | 0.022 | 0.049 | Yes | Yes |
| Overall matched cohort | Adequate relief in ≥50% of study weeks | Secondary effectiveness | χ² test | 0.050 | 0.076 | Yes | No |
| Overall matched cohort | ΔIBS-QOL at 8–12 weeks | Secondary effectiveness | Welch t-test | <0.001 | <0.001 | Yes | Yes |
| Overall matched cohort | IBS-QOL MCID responder, ≥14-point increase | Secondary effectiveness | χ² test | 0.021 | 0.049 | Yes | Yes |
| Overall matched cohort | Treatment escalation, any | Treatment-management outcome | χ² test | 0.036 | 0.062 | Yes | No |
| IBS-D subtype | ΔIBS-SSS at 8–12 weeks | Stratified effectiveness | Welch t-test | <0.001 | <0.001 | Yes | Yes |
| IBS-D subtype | IBS-SSS MCID responder | Stratified effectiveness | χ² test | 0.029 | 0.054 | Yes | No |
| IBS-D subtype | Pain ≥30% responder | Stratified effectiveness | χ² test | 0.010 | 0.041 | Yes | Yes |
| IBS-D subtype | Adequate relief | Stratified effectiveness | χ² test | 0.028 | 0.054 | Yes | No |
| IBS-D subtype | IBS-QOL MCID responder | Stratified effectiveness | χ² test | 0.016 | 0.049 | Yes | Yes |
| IBS-M subtype | ΔIBS-SSS at 8–12 weeks | Stratified effectiveness | Welch t-test | 0.002 | 0.010 | Yes | Yes |
| IBS-M subtype | IBS-SSS MCID responder | Stratified effectiveness | χ² test | 0.357 | 0.422 | No | No |
| IBS-M subtype | Pain ≥30% responder | Stratified effectiveness | χ² test | 0.696 | 0.696 | No | No |
| IBS-M subtype | Adequate relief | Stratified effectiveness | χ² test | 0.696 | 0.696 | No | No |
| IBS-M subtype | IBS-QOL MCID responder | Stratified effectiveness | χ² test | 0.516 | 0.583 | No | No |
| Baseline IBS-SSS <300 | ΔIBS-SSS at 8–12 weeks | Stratified effectiveness | Welch t-test | 0.009 | 0.041 | Yes | Yes |
| Baseline IBS-SSS <300 | IBS-SSS MCID responder | Stratified effectiveness | χ² test | 0.592 | 0.641 | No | No |
| Baseline IBS-SSS <300 | Pain ≥30% responder | Stratified effectiveness | χ² test | 0.284 | 0.351 | No | No |
| Baseline IBS-SSS <300 | Adequate relief | Stratified effectiveness | χ² test | 0.280 | 0.351 | No | No |
| Baseline IBS-SSS <300 | IBS-QOL MCID responder | Stratified effectiveness | χ² test | 0.274 | 0.351 | No | No |
| Baseline IBS-SSS ≥300 | ΔIBS-SSS at 8–12 weeks | Stratified effectiveness | Welch t-test | <0.001 | <0.001 | Yes | Yes |
| Baseline IBS-SSS ≥300 | IBS-SSS MCID responder | Stratified effectiveness | χ² test | 0.012 | 0.046 | Yes | Yes |
| Baseline IBS-SSS ≥300 | Pain ≥30% responder | Stratified effectiveness | χ² test | 0.039 | 0.064 | Yes | No |
| Baseline IBS-SSS ≥300 | Adequate relief | Stratified effectiveness | χ² test | 0.099 | 0.143 | No | No |
| Baseline IBS-SSS ≥300 | IBS-QOL MCID responder | Stratified effectiveness | χ² test | 0.023 | 0.049 | Yes | Yes |

Values are based on the propensity score-matched cohort. Raw P values for continuous outcomes were calculated using Welch’s t-test from the reported group means, standard deviations, and sample sizes. Raw P values for binary outcomes were calculated using χ² tests from the reported event counts and denominators. Benjamini–Hochberg false discovery rate adjustment was applied across the exploratory secondary, stratified, and treatment-management outcomes listed in this table. The primary continuous endpoint, ΔIBS-SSS in the overall matched cohort, was not included in this multiplicity-adjustment set because it was prespecified as the primary outcome. Safety outcomes were interpreted descriptively and were not included in this FDR adjustment table. IBS-SSS, Irritable Bowel Syndrome Symptom Severity Score; IBS-QOL, Irritable Bowel Syndrome Quality of Life; MCID, minimal clinically important difference; BH, Benjamini–Hochberg; FDR, false discovery rate.
